# Radio Signals from Live Cells: The Coming of Age of In-Cell Solution NMR

**DOI:** 10.1021/acs.chemrev.1c00790

**Published:** 2022-01-21

**Authors:** Enrico Luchinat, Matteo Cremonini, Lucia Banci

**Affiliations:** † Dipartimento di Scienze e Tecnologie Agro-Alimentari, Alma Mater Studiorum−Università di Bologna, Piazza Goidanich 60, 47521 Cesena, Italy; ‡ Magnetic Resonance Center, Università degli Studi di Firenze, Via Luigi Sacconi 6, 50019 Sesto Fiorentino, Italy; § Consorzio Interuniversitario Risonanze Magnetiche di Metallo Proteine, Via Luigi Sacconi 6, 50019 Sesto Fiorentino, Italy; ∥ Dipartimento di Chimica, Università degli Studi di Firenze, Via della Lastruccia 3, 50019 Sesto Fiorentino, Italy

## Abstract

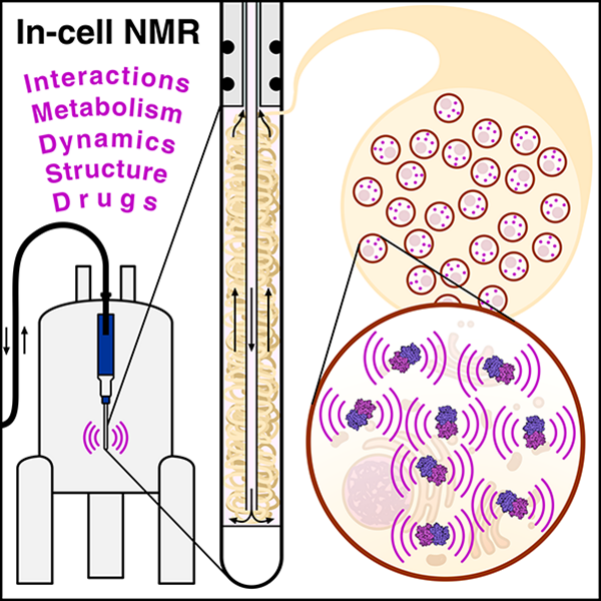

A detailed knowledge of the complex processes that make cells and organisms alive is fundamental in order to understand diseases and to develop novel drugs and therapeutic treatments. To this aim, biological macromolecules should ideally be characterized at atomic resolution directly within the cellular environment. Among the existing structural techniques, solution NMR stands out as the only one able to investigate at high resolution the structure and dynamic behavior of macromolecules directly in living cells. With the advent of more sensitive NMR hardware and new biotechnological tools, modern in-cell NMR approaches have been established since the early 2000s. At the coming of age of in-cell NMR, we provide a detailed overview of its developments and applications in the 20 years that followed its inception. We review the existing approaches for cell sample preparation and isotopic labeling, the application of in-cell NMR to important biological questions, and the development of NMR bioreactor devices, which greatly increase the lifetime of the cells allowing real-time monitoring of intracellular metabolites and proteins. Finally, we share our thoughts on the future perspectives of the in-cell NMR methodology.

## 1 Introduction

Progress in Medical Sciences and Life Sciences, in general, require detailed knowledge of the complex biological processes that underlie the function of a cell, the organization and interplay of multicellular structures, and, eventually, of the whole organism. Such a basic understanding has an enormous impact on our life, as it is necessary to understand diseases and to develop better drugs and therapeutic protocols. The cell, be it a pathogenic bacterium or a motor neuron, could be thought of as the fundamental unit of Life. However, a closer look at its inner workings reveals a hugely complex machinery, made up of a multitude of small and large molecules. Membrane proteins and lipids interact with each other in an orderly manner, to create larger structures—membranes—that segregate the inner aqueous solution in different compartments—organelles—and control the diffusion of soluble components in a selective manner. Other proteins dynamically organize as fibrils, making the cytoskeleton, that ultimately allow the cell to maintain its integrity and to control its shape and motility. Inside, DNA, RNAs, and the ribosomes take care of storing and translating the genetically encoded information, while other associated proteins regulate such processes and define the cellular phenotype. The intracellular aqueous compartments are filled with a plethora of soluble ions, metabolites, and macromolecules, which make up the intricated biochemical and signaling pathways that make the cell self-sustaining and ultimately “alive”.

Drawings of the interior of the cell, reconstructed at single-molecule detail, have become famous outside the field of Structural Biology, also thanks to the marvelous paintings by David S. Goodsell, his digital illustrations in the Protein Data Bank, and the openly available software for “cell painting”.^1−3^ These illustrations perfectly summarize the current knowledge of atomic-resolution structures of macromolecules,^4,5^ made possible by the development and application of X-ray crystallography, nuclear magnetic resonance (NMR) spectroscopy, and, more recently, single-particle cryo-electron microscopy (cryo-EM). However, currently, almost all the atomic-level studies of macromolecular structure, chemistry, and interactions have been obtained in vitro, extracting the molecules from the real environment of the living cell. Furthermore, while admirable, still pictures lack the time dimension and, therefore, cannot convey the idea that motions, from the picosecond to the year time scales, are at the basis of all processes of Life. Indeed, molecules making up the intracellular milieu move around in a (only apparently!) chaotic manner, undergo chemical and conformational changes, and interact with substrates, cofactors, and partners.

Of the above structural techniques, NMR spectroscopy is the only one able to obtain information on the structure, the kinetics, and the thermodynamics of biological macromolecules at the atomic level, as it can observe them in native-like environments at physiological temperatures, and it can do so in a nondestructive manner.^6^ Such a feature has always made NMR spectroscopy appealing for the study of small and large molecules not only in vitro, isolated from their physiological context, but directly inside intact living cells. Compared to other spectroscopic techniques, NMR suffers from an intrinsically low sensitivity; therefore, its applicability to cells was traditionally restricted to the observation of small, highly abundant molecules. Indeed, in the past century, cellular NMR studies were mostly focused on the analysis of cellular metabolism, for example, by exploiting the observation of phosphorus-containing molecules through ^31^P NMR, or by introducing ^13^C-labeled precursors for a metabolic flux analysis. In some cases, very abundant small macromolecules could be studied, often because of peculiar properties that made them stand out against the rest of the milieu, as it is the case for highly shifted signals of paramagnetic metalloproteins. Then, in the early 2000s, it became clear that modern NMR spectrometers, with a higher magnetic field and more sensitive hardware, could detect signals from isotopically labeled proteins inside the bacteria in which they were recombinantly expressed.^7^ Shortly after, macromolecules—proteins at first, then nucleic acids—were delivered to eukaryotic cells. The cellular NMR approach, reborn as “in-cell NMR”, soon gained widespread recognition, in a time when the scientific community had realized the importance of performing biochemical and biophysical studies in physiologically relevant contexts, and huge advancements were being made in developing techniques, such as single-molecule Förster resonance energy transfer (FRET) and cryo-electron tomography, that would be able to characterize macromolecules in a cellular environment.

This work provides a detailed overview of the development and applications of in-cell solution NMR approaches during the first ∼20 years since its inception in the modern sense. We first describe the existing approaches for cell sample preparation, the various types and strategies for isotopic incorporation, and the NMR methods that can be applied to living cells. We then review the application of in-cell NMR to different biological questions: how the cellular environment affects the folding thermodynamics of a protein, its structural and dynamic properties, and its interactions with specific cellular partners; whether the structure of a folded protein in cells differs from that determined in vitro; how proteins reach their mature, active state and how their redox state and post-translational modifications are regulated; the effect of the cellular environment on the conformational dynamics of intrinsically disordered proteins; how cell permeability and drug selectivity affect drug binding to an intracellular target; the properties of nucleic acid structural motifs and their interactions with drugs and other compounds. Finally, we provide an overview of NMR bioreactor devices, which allow to greatly increase the lifetime of the cells in the NMR spectrometer, and their applications to monitor intracellular metabolism, protein–ligand/protein–protein interactions, and chemical modifications in real time. To ensure that each section can be read independently, works that report both methodological advancements and application to biological systems may be referenced multiple times across the text. Finally, in the last section we summarize the current strengths and weaknesses of in-cell solution NMR, and we share our vision for the future development of the methodology toward its application to more challenging and physiologically relevant systems.

## 2 Methodological Aspects

### 2.1 Sample Preparation

The successful detection of intracellular macromolecules by solution NMR spectroscopy requires that the molecules of interest are (1) free to tumble within the cell, (2) present at a sufficiently high concentration to overcome the low sensitivity of the technique, and (3) observed without too strong interference from other cellular components. While the first aspect is an intrinsic limitation of solution NMR, meeting the other two requirements is possible but poses additional challenges to the way cell samples are prepared. Indeed, sample preparation has become a central aspect in the development of in-cell NMR methods, and choosing the most appropriate approach for a given application is strategic for ensuring both the success of the experiments and the biological relevance of the data obtained.

**Figure 1:**
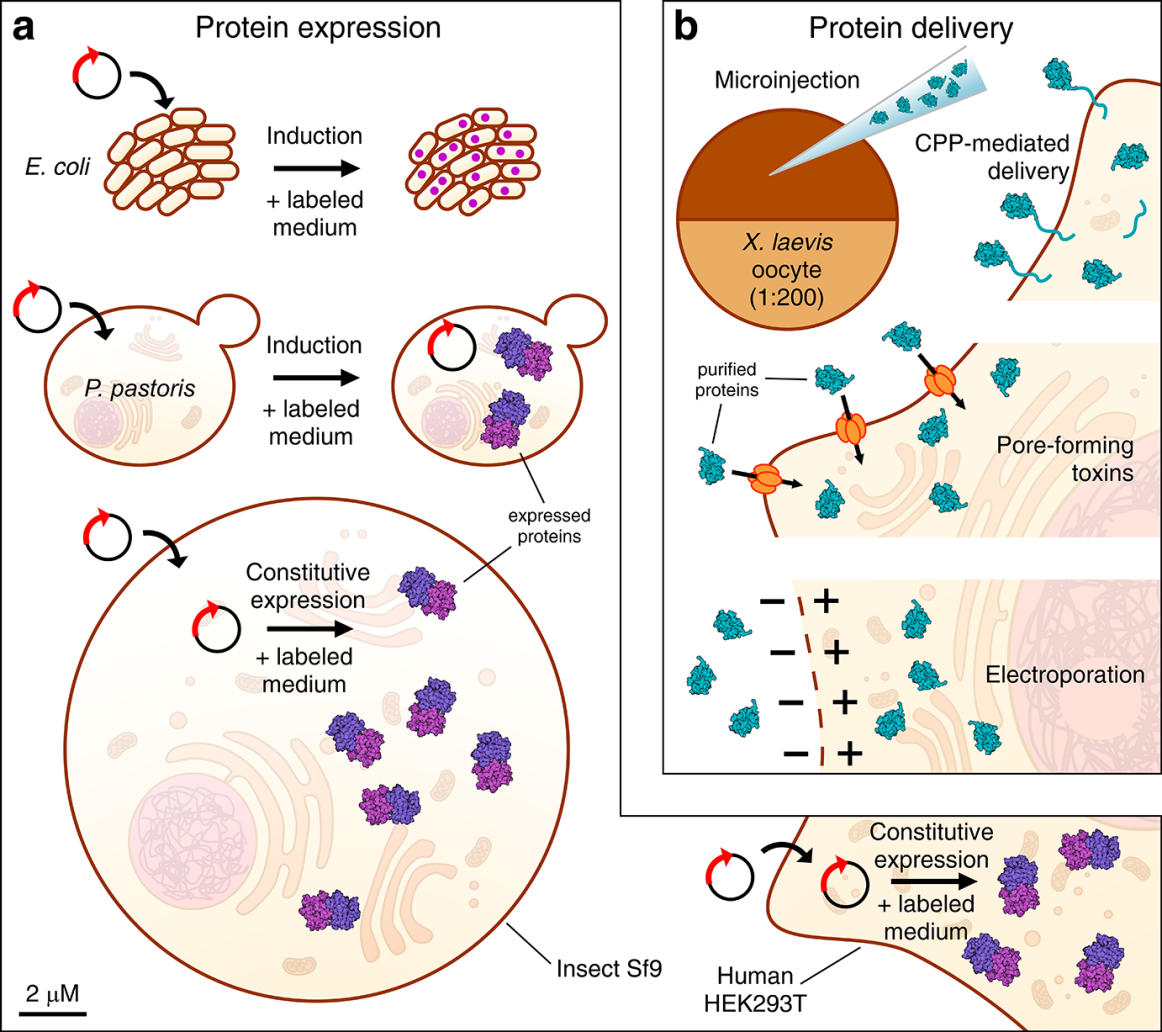
Overview of the different approaches for in-cell NMR sample preparation. (a) Proteins (violet) can be directly expressed in the cells to be analyzed by NMR: bacteria and yeast cells (top, middle) are transformed with an inducible expression vector; insect cells (bottom left) are infected with a baculoviral vector; human cells (bottom right) are transfected with a constitutive expression vector. Protein expression is performed in isotopically enriched media. (b) Recombinantly expressed and purified proteins (teal) or nucleic acids (not shown) can be microinjected into *X. laevis* oocytes (top left) or delivered to human cells by using CPP-fused constructs (top right), by permeabilizing the cells with pore-forming toxins (middle) or by electroporating the cells (bottom). All cell types (but not proteins) are drawn approximately to scale; the oocyte is scaled down by 1:200.

The approaches developed up to now can be roughly classified in two main lines: one exploits the expression of the protein of interest directly in the cells of choice (Figure 1a), either through transformation (for bacteria and yeast) or infection/transfection (for insect/mammalian cells, respectively). The other relies on the use of different delivery approaches to introduce purified proteins or nucleic acids, previously expressed by other types of cells or chemically synthesized, into the selected cells (Figure 1b). The use of one approach rather than the other depends on the type of molecule under study and on several other factors. Indeed, for DNA/RNA molecules, the delivery of a purified molecule is the only approach demonstrated so far. If proteins are to be investigated, the choice depends on various factors, from the type of cells in which the protein is studied, to whether chemical modifications (e.g., incorporation of non-natural amino acids or conjugation with tags) or isotope-labeling schemes (e.g., amino acid-selective labeling, deuteration) are needed. In addition, choosing the best approach when applying in-cell NMR to study a protein for the first time also depends on protein-dependent factors (e.g., protein stability, expression efficiency, toxicity etc.), which are often unknown a priori and must be evaluated.

#### 2.1.1 Protein Delivery

The early examples of protein in-cell NMR spectroscopy relied upon protein expression and direct observation in the host cells and, as such, were “limited” to the most widely used, versatile prokaryotic expression system, *Escherichia coli*. In the early 2000s, eukaryotic expression systems (with the exception of yeast) were still poorly developed. Therefore, protein delivery approaches were first developed to allow NMR studies of proteins in eukaryotic cells. In these approaches, the protein of interest is produced recombinantly (usually in *E. coli*), purified, and subsequently introduced into the desired cells by either physical or (bio)chemical methods. In addition to solving the issue of reaching NMR-detectable levels of protein in eukaryotes, protein delivery offers another advantage compared to expression: isotope-labeled recombinant proteins are introduced in an unlabeled cellular environment, thus eliminating (or at least greatly reducing) the interference arising from the cellular background signals during the acquisition of heteronuclear NMR spectra.

Protein delivery was first employed to observe proteins by NMR in African claw-frog (*Xenopus laevis*) oocytes in two concurrent works by the Shirakawa and Wagner groups, where labeled proteins were introduced through the use of a mechanical microinjection procedure (Figure 1b).^8,9^*X. laevis* oocytes have approximately a 1 mm size in diameter that makes them easily manageable and suitable for the application of this kind of approach. Conventional protocols are available for the oocytes extraction and preparation.^10−12^ After an extraction from mature adult females, the oocytes are examined under the microscope, where the healthy ones are selected on the basis of morphology and pigmentation. Oocyte cells stored at 18 °C in the appropriate buffer can be maintained in a healthy condition for several days.^10^ Approximately 150–200 oocytes are needed for an NMR experiment. The microinjection procedure is generally performed manually or by the use of automatic microinjectors.^8,9^ The precise control of the amount of protein injected is one of the main advantages of the method, which ensures an excellent sample homogeneity. Oocytes are also employed for the extraction of a crude lysate with minimal dilution of the cell content. This is widely used to simulate the cell cytoplasmic fraction and to study molecules or biological processes^13,14^ within an environment in which viscosity and macromolecule compositions are similar, to some extent, to those of intact cells. In addition to *X. laevis*, recently *Danio rerio* oocytes have been shown by the Pielak group to be amenable to in-cell NMR spectroscopy.^15^

While the oocyte microinjection is a versatile approach, the procedure may be time-consuming if a large number of samples is to be prepared, and it requires an injection of a small volume of a highly concentrated protein solution, which could limit the final intracellular concentration in the case of aggregation-prone macromolecules. Furthermore, oocytes isolated from different individuals and seasonal variability could affect the reproducibility of the method.^16^

A few years after the first in-cell NMR experiments on eukaryotic cells were reported, Shirakawa published the first NMR observation of proteins delivered in cultured human cells, by employing a biochemical delivery method mediated by cell-penetrating peptides (CCPs).^17^ This technique had been developed and successfully adopted to introduce different active biomolecules inside living cells and has since received much attention for its promising applications to biological drug delivery.^18−20^ The approach exploits the ability of short positively charged peptides, which can be either naturally occurring or designed specifically, to promote protein internalization directly into the target cells (Figure 1b). Among the available delivery vectors, Inomata et al. employed the fragment comprising the residues between 47 and 58 (-YGRKKRRQRRR-) of the human immunodeficiency virus 1 (HIV-1) Tat protein,^21^ which is still one of the most used. The CPP can be either introduced recombinantly with the cargo protein as a fusion construct or chemically conjugated after purification. When the cells have been incubated with the CCP–protein adduct, the internalization is accomplished usually in 15–30 min.^22^ Notably, however, two different translocation mechanisms of the protein inside the cells have been reported: either through a direct translocation or through endocytosis.^23^ Through the latter process, the protein localizes inside endosomes and does not reach the cytosol. To avoid an endosomal localization, the direct translocation mechanism can be promoted by incubating the cells with the CCP–protein system in the presence of pyrenebutyrate.^24^ The electrostatic interaction between the CPP, which is arginine-rich, and pyrenebutyrate, which is negatively charged and harbors an aromatic moiety, decreases the net charge and increases the net hydrophobicity of the complex, facilitating its direct translocation across the lipid bilayer of the plasma membrane.^24−26^ On the one hand, this method has proven to be highly effective for protein delivery and can be applied to a variety of human cell lines, primary cultured cells, and even multicellular organisms.^21,23,27,28^ On the other hand, the translocation efficiency of a CCP-fused protein is highly dependent on the surface charge distribution of the protein to be delivered, its hydrophobicity, and likely on other physicochemical properties. This limits the applicability of CPP-mediated delivery to in-cell NMR, where high protein levels are required, unless the surface properties of the protein of interest are modified—sometimes heavily—to increase its delivery efficiency.^29^ Partly as a consequence of this limitation, a CPP-mediated delivery for in-cell NMR has been successfully applied to few proteins: ubiquitin-3A mutant and FKBP12,^17^ calbindin D_9k_,^30^ and the superoxide dismutase 1 (SOD1) β-barrel (SOD1^ΔIVΔVII^), that is, a truncated form of human SOD1 lacking the loops IV and VII.^29^

A different delivery technique relies on the use of a bacterial toxin, streptolysin O (SLO), which fuses with the plasma membrane of mammalian cells to form 35 nm wide pores, large enough to allow exogenous molecules up to ∼150 kDa to translocate and reach the cytosol (Figure 1b).^31^ The pore formation mechanism can be reversed by adding Ca^2+^ after protein internalization. This method has been successfully employed to introduce small amounts of different molecules like antigens^32^ or oligonucleotides^33^ inside adherent and nonadherent cellular types. Its first application to in-cell NMR was demonstrated by Shimada and co-workers, who delivered thymosin β-4 in human cells at sufficient concentrations for NMR detection.^34^ This technique allows one to introduce NMR-compatible concentrations of proteins inside a broad range of living cells without resorting to modifications of the target protein, as opposed to a CPP-mediated delivery. However, some leakage problems were reported by the authors in the above work, where propidium iodide staining revealed the presence of some unrepaired pores after the Ca^2+^-mediated resealing, with a consequent leakage of the molecules of interest that could give rise to unwanted NMR signals.^34^ Therefore, the experimental conditions for the treatment with SLO and subsequent cell recovery must be carefully tuned. Nevertheless, an SLO-mediated protein delivery was later successfully applied to observe other proteins by NMR in human cells, namely, CAP-Gly1 (a small microtubule-binding domain),^35^ thioredoxin,^36^ and the GTPase domain of HRAS.^37^

An alternative protein delivery approach, which makes use of electroporation (EP), was proposed by Selenko and co-workers with the aim of expanding the applicability of in-cell NMR spectroscopy to a wider set of cell lines, which would allow the study of specific biological processes in a more physiologically relevant cellular context.^38−40^ The technique was originally developed to introduce exogenous nucleic acids into cell lines that would be hard to transfect via classical methods.^41,42^ It was later demonstrated that other types of molecules could be introduced with the same principle.^43^ The EP process consists in the application of short and strong electric pulses to a cell suspension. This leads to the formation of transient cracks on the plasma membrane, thus allowing the migration of external molecules in the cellular cytoplasm (Figure 1b). By varying the pulse length, power, and timing, the technique can be extended to several types of cells, both eukaryotic and prokaryotic, therefore making EP a very versatile tool for in-cell NMR.^44^ However, many parameters need to be optimized to maximize the EP efficiency in terms of percentage of electroporated cells, amount of protein delivered, and preservation of cell viability. Given the chemical complexity of living cells, it is nearly impossible to predict the optimal values for different types of cells. Therefore, in practical terms, such a careful optimization of the experimental conditions must be done empirically for every single cell type, making the approach time- and resource-intensive. Even worse, when electroporating proteins, the efficiency of delivery appears to be very protein-dependent. Indeed, similarly to a CPP-mediated delivery, the protein folding state and its surface properties can strongly affect its behavior as it interacts with the permeabilized plasma membrane. The strong electric field applied can also negatively affect the protein, depending on its net charge and surface distribution, and could even cause its unfolding,^45^ although this latter issue clearly does not arise when intrinsically disordered proteins (IDPs) are investigated, as in the case of α-Syn.^38,39^ Despite its limitations, EP has found further application across the in-cell NMR scientific community. Besides α-Syn, which remains the “golden standard” for EP-based protein delivery, EP has since been employed to deliver other proteins, both folded and unfolded, into cultured human cells for NMR studies: SOD1 β-barrel,^46^ wild-type ubiquitin,^47,48^ ubiquitin-3A conjugated to a lanthanide tag for paramagnetic NMR applications,^49^ adenylate kinase 1,^50^ and two different isoforms of Tau, namely, Tau40 and k19.^51^ Furthermore, EP can also be employed to deliver nucleic acids to cultured cells for NMR applications (nucleic acid delivery by EP is discussed in more detail in section 2.1.3).

Finally, while out of the scope of this Review, it is worth mentioning that in-cell electron paramagnetic resonance (EPR) spectroscopy is rapidly emerging as a promising approach complementary to NMR to obtain site-specific insights into the internal dynamics and long-range distances in macromolecules in living cells.^52,53^ EPR measurements require a site-directed spin labeling of the studied molecules followed by an intracellular delivery, and the EPR community is currently developing suitable paramagnetic tags and EP protocols to deliver spin-labeled proteins in bacteria and mammalian cells for in-cell EPR applications, following the first example provided by Selenko and Goldfarb.^39^ Besides EP, thermal shock has recently been shown to be effective for a tagged protein delivery for EPR studies.^54^

#### 2.1.2 Protein Intracellular Overexpression

Alongside delivery systems, the intracellular expression of recombinant proteins proved to be a valid strategy for an in-cell NMR sample preparation. Unlike the protein delivery methods, the intracellular expression allows one to study the proteins directly in the cell where they are produced. In this way, the expression and purification steps are avoided; thus, the “DNA-to-NMR” workflow, that is, the series of steps required to obtain a sample of cells for NMR starting from the DNA encoding the protein of interest, is generally shorter when compared to protein delivery approaches. On the one hand, a direct expression is also advantageous when dealing with proteins particularly susceptible to hydrolysis or oxidation or that are prone to aggregation. On the other hand, the advantages of direct expression come at the cost of a decreased isotope labeling selectivity and a much more limited toolset of chemical modifications and, for eukaryotes, of protein labeling schemes. Indeed, the direct expression in the cells to be observed implies that these are grown in isotope-enriched media. As the cells grow and metabolize isotope-enriched nutrients, other cellular components will be isotope-labeled in addition to the protein of interest. Moreover, chemical modifications such as conjugation with fluorophores or spin labels are not possible; in eukaryotic expression systems, the use of isotope-labeled precursors is more limited, and ^2^H enrichment is severely hampered by toxic effects. Nevertheless, many prokaryotic and eukaryotic expression hosts have been employed for in-cell NMR.

Among prokaryotic organisms, *E. coli* is by far the most commonly used, while, among eukaryotes, direct expression systems for yeast, insect, and human cells have been developed. *E. coli* is undoubtedly the best known and studied bacterial strain. Given the ease of manipulation and the variety of existing vectors and protocols for a recombinant protein expression, *E. coli* has been extensively employed in the field of in-cell NMR.^7,55−59^ The sample preparation strategies are substantially similar to typical protein expression protocols, in which the strain of choice is transformed with a vector encoding the gene of interest, usually induced by isopropyl β-d-1-thiogalactopyranoside (IPTG). To increase the isotope labeling selectivity of the expressed protein versus cellular background, the cells are grown in an unlabeled medium prior to induction, which is then replaced with a labeled medium during protein expression (Figure 1a). Cells are then collected and suspended in the NMR tube as a thick cell slurry for the NMR analysis.^7,60^ An advantage of protein expression in bacteria over other cell types is the high cell densities reachable in a few hours and the low cost of isotope labeling when minimal media are used. However, the bacterial protein synthesis machinery does not allow the possibility of studying more complex systems. In fact, *E. coli* is unable to perform post-translational modifications, such as glycosylation and lipidation, which are often important for the function of many human proteins. More generally, it is often desirable to investigate proteins in a cellular environment as close as possible to the physiological one, especially when NMR is used to gain an atomic-level insight on the functional aspects of a protein. Therefore, when studying eukaryotic proteins, a matching eukaryotic expression host is required.

Among eukaryotic microorganisms, the yeast *Pichia pastoris* is the most commonly used for protein expression, due to its ability to reach extremely high cell densities and to produce high amounts of intracellular or secreted proteins, and also for the availability of multiple strong and tightly regulated promoters. As such, it has been the ideal workhorse for protein production, even on an industrial scale, since the 1980s.^61,62^ Its alcohol oxidase promoter (P_AOX1_) is strongly repressed when *P. pastoris* grows in the presence of glucose, glycerol, or ethanol as carbon sources.^63^ Instead, when methanol is added to the culture medium, P_AOX1_ is fully induced. Despite these premises, *P. pastoris*—and yeast generally—has been rarely used for in-cell NMR applications, compared to *E. coli* and to other expression hosts. The first example of the use of this expression host for in-cell NMR observation dates back to 2012, when the Shekhtman group employed *P. pastoris* to express ubiquitin and investigate how the cellular metabolic state influenced its structure and cellular localization (Figure 1a).^64^ Yeast ubiquitin was expressed in a P_AOX_-controlled manner. Protein expression was triggered by replacing the medium with two different methanol-containing media, and the effects of the resulting metabolic states on the protein localization and tumbling were investigated by NMR and fluorescence microscopy.^64,65^ The yeast *Saccharomyces cerevisiae*, currently, has been used for in-cell NMR only in one case, by Wall and Hough, who investigated the conformational dynamics of the FG repeats-containing nucleoporin Nsp1 and its interactions within the bacterial and yeast cytosol.^66^

Overall, yeast has proven to be an easy handling and cost-effective expression system for both laboratory research and large-scale protein production. However, the difficulties encountered when expressing in yeast heterologous proteins requiring more complex post-translational modifications^67^ have prompted scientists to explore new approaches. Among the eukaryotic expression systems alternative to yeast, a baculovirus-mediated insect cell expression system was first described in the early 1980s,^68^ and since then, important technological improvements have contributed to make it one of the most effective and intensively used methods for eukaryotic recombinant protein expression. Insect cells contain molecular chaperones more similar to those of human cells, and their protein processing machinery allows the correct folding of more complex proteins and a series of post-translational modifications that would have been impossible in bacteria and yeast.^69,70^ The insect cell lines Sf9 and Sf21, derived from the fall armyworm *Spodoptera frugiperda*, are the most commonly used as expression systems.^70^ In the baculovirus expression vector system (BEVS), first the gene encoding polyhedrin, a protein that is produced in a large amount at the final stage of the viral infection, is replaced by the target protein gene, and the engineered virus is amplified. While this procedure must be repeated every time a new construct is to be tested, making it somewhat cost- and time-intensive, nowadays commercially available systems make baculoviral vector production relatively straightforward.^71^ Cultured insect cells are then infected with the engineered virus, and the protein expression starts within a few hours after the internalization (Figure 1a).^69,70^ The application of this approach for protein NMR studies became possible with the development and commercial availability of isotope-labeled media for insect cell cultivations and with the possibility to introduce specific isotope-labeled amino acids.^70^ The first example of in-cell NMR in insect cells was reported by Shirakawa and Ito in 2013.^72^ In that work, four different proteins were expressed and labeled in Sf9 cells and detected by in-cell NMR: *Streptococcus* protein G B1 domain (GB1), *Thermus thermophilus* HB8 TTHA1718, rat calmodulin (CaM), and human HAH1. The expression was performed for 48 h, a substantially longer time that those required for protein expression in bacteria and yeast. By exploiting the lag time between infection and expression, the isotope-labeled medium could be provided 24 h postinfection with a minor decrease in the labeling efficiency of the expressed proteins, thus reducing the cellular background in the NMR spectra. The background was further suppressed during spectral processing, by subtracting a spectrum of insect cells infected with an empty vector. The use of this expression system was demonstrated to be suitable for heteronuclear multidimensional in-cell NMR and was later shown by the same authors to make possible a nuclear Overhauser effect (NOE)-based protein three-dimensional (3D) structure determination in insect cells.^73^ In principle, a baculoviral expression also provides a way for characterizing, by in-cell NMR, complex proteins that cannot be processed correctly in lower organisms. Although the high cost of labeled culture media could represent an issue for the broad applicability of the method, the initial expense is mitigated by the fact that very small culture volumes (5 mL) are required for each NMR sample.^72^ Furthermore, less expensive isotope-labeling methods using algal or yeast extracts could be employed.^74^

One of the goals of in-cell NMR is to investigate proteins and their molecular processes, such as maturation, folding, and interaction with partners directly within the cellular milieu. Therefore, for human proteins, the ideal cellular environment is obviously that of human cells. Traditionally, the use of mammalian cells as expression hosts is considered costly and time-consuming. Cell cultures need to be transiently transfected every time with high amounts of DNA, a procedure that requires sophisticated reagents to allow DNA uptake, is not always efficient, and can be toxic to the cells. In addition, the protein levels reached with the commonly used mammalian expression vectors and cell lines are often too low to allow NMR detection. In the early 2000s, the human embryonic kidney (HEK) 293EBNA was one of the first human cell lines to be efficiently transfected and employed to produce secreted proteins on a large scale.^75−77^ Starting from those studies, a series of HEK293 derivative cell lines and different expression vectors were developed. A fast and cost-effective mammalian expression system for the high-yield expression of secreted proteins was proposed by the Jones lab.^78^ The pCAβ-EGFP plasmid, derived from the pCAGGS plasmid,^79,80^ was chosen as an ideal scaffold for the vector design. It contains a CAG promoter, one of the strongest synthetic promoters, for a high-level constitutive protein expression.^81^ The resulting vector, pLEXm, was then modified to obtain a series of plasmids that included the presence of different purification and detection tags as well as multiple cloning sites and secretion sequences. A cheap, efficient transfection of HEK293T with low toxicity was achieved with a polyethylenimine (PEI)-mediated protocol.^78^ In parallel, the advent of commercially available uniformly labeled media for mammalian cells had made it possible for one to express and isotope-label challenging proteins in human cells for NMR applications.^82,83^ These advancements made possible protein expression in human cells for in-cell NMR.^84,85^ The Banci group adapted the system developed by Aricescu et al. to express isotopically labeled proteins in HEK293T cells at sufficient levels for NMR detection (Figure 1a).^84,85^ The pHLsec plasmid, originally built from the pLEXm to express secreted proteins,^78^ was reverted to a cytoplasmic expression vector by removing the N-terminal secretion signal sequence. The sample preparation, described in detail elsewhere,^85^ is similar to that reported for protein expression and labeling in insect cells and conceptually analogous to all other protein expression approaches: cells are first grown as a monolayer in unlabeled medium and then transiently transfected (via DNA:PEI complexes) with the vector encoding the protein of interest, and the medium is replaced with the isotope-labeled one. Protein expression occurs in the 48 h following transfection, followed by cell collection and NMR analysis. Similar to insect cells, the spectral background arising from the nonspecific labeling of cellular components can be greatly reduced when processing the spectra, by subtracting a spectrum of human cells transfected with an empty vector.^85^ The PEI-mediated transfection ensures a high plasmid copy number per cell and makes it straightforward to simultaneously coexpress two proteins (or more, in principle), ensuring that each cell incorporates both genes,^84^ thus allowing the NMR observation of intracellular protein complexes.^86^ By the same principle, the intracellular protein levels can be decreased at will by “diluting” the vector encoding the protein of interest with an empty vector. Following the first application of this approach to monitor the maturation of wild-type SOD1,^84^ the Banci group successfully studied by NMR several proteins expressed in the cytoplasm of HEK293T cells: a set of SOD1 mutants linked to familial Amyotrophic Lateral Sclerosis (ALS),^87^ the copper chaperone for SOD1 (CCS, both the full-length and the SOD-like domain),^84,86^ two small mitochondrial proteins, Mia40 and Cox17, prior to their import in the mitochondrial intermembrane space (IMS),^88,89^ the actin-binding protein profilin 1,^90^ HAH1,^84^ the deglycase DJ-1,^91^ and the isoforms I and II of carbonic anhydrase.^92^ Furthermore, the same group showed that proteins fused to an N-terminal targeting sequence expressed in human cells could be targeted to the mitochondrial IMS, allowing one to observe their isolated intact mitochondria by in organello NMR.^93^ As stated above, the transient transfection allows the coexpression of two or more proteins, which is useful for studying protein–protein interactions. However, in this way both proteins are identically labeled, which is undesirable in NMR due to the severe signal overlap in the resulting spectra. To overcome this limitation, Luchinat and coauthors proposed a variation of the approach, in which two proteins are expressed in a sequential manner by combining stable and transient expression.^94^ In that work, human HEK293T cells were stably transfected with a plasmid encoding HAH1 under a constitutive promoter and containing the PhiC31 integrase gene.^95^ Then, the cells were cotransfected with two plasmids, one encoding SOD1 under a constitutive promoter, the other containing a short hairpin RNA responsible of silencing the HAH1 expression by RNA interference (RNAi). The selective ^15^N-labeling of SOD1 was achieved by a proper timing of the incubation with the labeled medium. While working in principle, this approach did not find a practical application owing to the much lower expression level achieved by the stable cells and to the lack of expression control of the stable protein.

Overall, the direct protein expression in human cells is a promising approach that can be complementary to protein delivery. The expression system, although restricted to HEK293T cells, is generally robust when applied to small- and medium-sized soluble proteins, whereas protein delivery is applicable to more cell lines, but its efficiency is highly protein-dependent and must be carefully optimized. Arguably, time- and cost-wise, a direct protein expression is advantageous: despite the high cost per liter of uniformly labeled media for mammalian cells, like with insect cells, each NMR sample requires a small amount of medium (20 mL), and there is no need for a large-scale protein purification and delivery, significantly cutting the sample cost and preparation time. On the one hand, cheaper media preparations such as algal autolysate-based labeled media can also be employed to further reduce the costs.^96^ On the other hand, the higher labeling selectivity makes protein delivery more appealing spectroscopy-wise, as it provides background-free NMR spectra and increased sensitivity at low protein concentrations (i.e., when the signal-to-noise ratio is higher than the signal-to-background ratio), especially when observing signals from IDPs, that are highly overlapped with the cellular background.

#### 2.1.3 Nucleic Acids Delivery

The structure and dynamic properties of nucleic acids, such as DNA and RNA, can be very sensitive to the molecular environment. Indeed, it is known that the conformation of some DNA/RNA motifs changes dramatically in vitro, as a function of pH, ionic strength, and the presence of specific counterions.^97,98^ Furthermore, crowding and interaction partners also affect nucleic acid conformations and dynamics.^97,99^ Therefore, in-cell NMR represents an ideal technique to study the conformation and interactions that nucleic acids establish with intracellular molecules. The approaches used for the delivery of the exogenous nucleic acid fragments of interest directly into the living cells are essentially the same as those described above for protein delivery. Because of the technical challenge of successfully inserting nucleic acids in cultured cells, until three years ago the only cells used as targets for nucleic acids delivery were *X. laevis* oocytes. Similar to proteins, a highly concentrated stock solution of nucleic acid (e.g., ∼50 nl of an ∼3 mM solution) is microinjected in each oocyte.^100^ This method also allows the study of interactions, where both DNA/RNA and a possible partner are coinjected. Similar to proteins, a limitation of the oocyte injection is the requirement of a highly concentrated external solution, which can lead to oligomerization and aggregation processes.^101^ Unlike proteins, however, nucleic acids, especially RNA, suffer from an additional drawback: they often have a short half-life in the intracellular environment, where they are quickly hydrolyzed by intracellular nucleases. In the first example of in-cell NMR of nucleic acids in oocytes by the Trantirek and Schwalbe groups,^102^ the rate of hydrolysis and other critical parameters for NMR were evaluated. It was found that the time needed for the injection in all the needed 150–200 oocytes, especially if done manually, can be very close to the intracellular half-life of the injected nucleic acids. A chemical modification in the DNA backbone by replacing the first two phosphate groups with phosphorothioate groups resulted in a higher resistance to the nuclease-mediated degradation. RNA can also be stabilized by introducing the same modifications in addition to the methylation of the O2′-hydroxyl group.^102^ Furthermore, particularly stable RNA secondary structures can improve its resistance to degradation within the cell. Recently, Trantirek and Schwalbe showed that an ∼70 nt-long RNA aptamer delivered to oocytes with no chemical modifications was sufficiently stable to allow one to perform an in-cell NMR analysis over a course of ∼15 h.^103^

As with proteins, human disease-related nucleic acid motifs should be ideally studied in human cells, which can provide more physiologically relevant insights with respect to frog oocytes. In recent years, two delivery approaches, previously exploited for proteins, were applied to deliver nucleic acids into human cells. Yamaoki et al. showed that DNA and RNA could be delivered to HeLa cells by using the pores-forming toxin SLO.^104^ The procedure is similar to the one used for proteins: HeLa cells permeabilized with SLO were incubated with nucleic acids, and then calcium chloride was added to reseal the pores. The nucleic acid localization was assessed to be mainly in the nucleus, and the intracellular concentration was estimated between 5 and 20 μM.^104^ Similar to proteins, during the incubation with SLO, leakage of the intracellular content occurred due to an estimated 35–40% cell mortality rate. To overcome this problem, SLO-treated cells were incubated with a solution containing a cytosolic extract together with adenosine triphosphate (ATP), creatine kinase, and creatine phosphate. This treatment improved the recovery after the pore sealing, resulting in a higher survival rate. In addition, to remove the remaining dead cells, a Percoll gradient centrifugation was employed as a final step prior to the NMR investigation.^104^

At the same time, the Trantirek group exploited electroporation for the delivery of nucleic acids into HeLa cells for in-cell NMR.^105^ Similar to the procedure for proteins, a cell suspension is subjected to a series of electric shocks, alternating high and low voltages with periods of rest, which cause the formation of cracks on the cell membranes, allowing the nucleic acids, present at a concentration of 300–400 μM in the electroporation buffer, to enter inside the cells. The perturbed plasma membrane is then able to seal spontaneously a few minutes after the treatment, and a nucleic acids intracellular concentration of 5–20 μM is reached. Compared to protein electroporation, the nucleic acid delivery suffers from less sample-dependent effects. This is likely due to the overall more similar electrostatic properties of different nucleic acid sequences, whereas proteins show a much higher variability. Further advantages of this method are the short time before starting the spectra acquisition and the high efficiency of insertion, ∼90%. Furthermore, the cell viability was estimated in the range of 80–95%, and no leakage was observed during the electroporation procedure.^105^ In the above work, the approach was applied to investigate the structural stability of DNA i-motifs in the nucleus by in-cell NMR. The Trantirek group further applied the electroporation approach to observe intracellular DNA–ligand complexes in human cells.^106^

### 2.2 Isotopic Labeling

Biomolecular NMR spectroscopy typically relies on spin-1/2 nuclides of biologically abundant atoms: ^1^H, ^13^C, and ^15^N. Given the low natural abundance of ^13^C (1.1%) and ^15^N (0.4%) isotopes, the molecules of interest must be isotopically enriched for the NMR analysis. This poses further requirements when preparing samples for in-cell NMR. Besides enabling heteronuclear experiments, isotope enrichment serves an additional purpose when investigating macromolecules in cells. In vitro, molecules are normally studied as pure substances, and thereby their NMR spectra do not contain interferences from other components. Instead, the complex mixture of the cellular milieu gives rise to unwanted NMR signals that, in the case of ^1^H (99.98% abundant), result in an extremely crowded NMR spectrum. Therefore, isotopic labeling also acts as a filter to eliminate background signals when investigating macromolecules in cells by exploiting the low natural background of ^13^C and ^15^N.

For proteins recombinantly expressed in *E. coli*, either for a direct in-cell NMR analysis or for purification and delivery into eukaryotic cells, several labeling schemes have been employed. In the initial studies by the Dötsch group, uniform ^15^N and selective [^15^N]lysine labeling were employed to compare the contribution of protein signals and cellular background in the heteronuclear two-dimensional (2D) spectra and the resulting spectral complexity.^7,60^ The same group showed that uniform ^13^C labeling results in highly crowded spectra, due to the presence of many highly abundant ^13^C-containing metabolites, whereas selective [^13^Cε]methionine labeling results in well-resolved, almost background-free spectra.^107^ In those studies, it became evident that a “medium switch” strategy—in which cells are first grown in an unlabeled medium up to the optimal density for induction, while protein expression is performed in a labeled medium—was necessary to reduce the cellular background. In the following years, uniform ^15^N labeling by a medium switch became the most widely applied labeling strategy for in-cell NMR studies of protein conformation, dynamics, and interactions. Well-resolved protein spectra with low background interference can also be obtained by recording 2D ^13^C-detected C–N correlations on uniform ^13^C,^15^N-labeled cell samples.^108^ More complex labeling schemes are required to perform a side-chain assignment and measure intramolecular NOEs for an in-cell protein structure calculation. For that purpose, Sakakibara and coauthors employed labeling strategies that resulted in different combinations of alanine, leucine, and valine residues with selectively protonated, ^13^C-enriched side-chain methyl groups in a uniform ^2^H,^15^N-labeled background, by using deuterated minimal media containing ^15^NH_4_Cl, [3-^13^C]alanine, and [U–^13^C, 3-^2^H]α-ketoisovalerate.^56^

A reduced proton density (REDPRO) labeling scheme,^109^ resulting in a side-chain specific protonation in a ^2^H-background, was employed by Shekhtman to improve transverse relaxation times of ^15^N-labeled proteins strongly interacting with cellular components, either directly observed in bacteria or purified and delivered to human cells.^47^ For the same purpose, the Shimada group delivered, into human cells, a protein selectively labeled with the ^13^C Ileδ1 methyl group in a ^2^H background.^37^

When proteins are directly expressed in insect or human cells, isotopic labeling cannot be performed in minimal media. Uniformly labeled media for cultured cells are commercially available for both type of cells, containing all the labeled essential amino acids and other metabolites required for cell growth at defined concentrations. These media preparations have been employed to express uniformly ^15^N- or ^13^C,^15^N-labeled proteins in insect and human cells for in-cell NMR.^72,84^ When labeling proteins in these cell types, cells are first grown in unlabeled media, and a medium switch is performed after the infection/transfection. The long expression times (∼48 h) cause the extensive labeling of other cellular proteins, giving rise to strong cellular background signals in the spectra, which can be decreased by shortening the labeling time window (e.g., by delaying the medium switch by a few hours after infection) and/or by performing background subtraction (see section 2.1.2). To reduce the spectral complexity, amino acid-type selective labeling can be performed with relative ease in these cell types, owing to the lack of synthetic pathways that would cause isotopic scrambling and incorporation in other amino acids. For protein structure determination in insect cells, the Ito group employed custom-made media supplemented with the desired combination of up to eight different ^13^C,^15^N-labeled amino acids.^73^ In human cells, the Banci group introduced selectively labeled amino acids in the expressed proteins by using custom-made media supplemented with [^15^N]cysteine, [^13^Cε]methionine, or [^15^N]histidine.^85,110^

Unlike proteins, nucleic acids are synthesized in vitro and delivered to the desired cells for NMR analysis. Therefore, with the proper synthetic procedure, nucleic acids can be labeled either uniformly or at specific positions. Uniformly ^15^N- or ^13^C,^15^N-labeled DNA and RNA have been shown to provide clean ^1^H–^15^N and ^1^H–^13^C spectra in oocytes by Dötsch/Trantirek and Mergny groups.^102,111^ Recently, an RNA aptamer in a complex with ^15^N-labeled 2′-deoxyguanosine was observed by one-dimensional (1D) ^15^N-edited and 2D ^1^H–^15^N spectra.^103^ Currently, the delivery of ^15^N or ^13^C,^15^N-labeled nucleic acids to mammalian cells is deemed impractical due to the high cost of the labeled precursors; therefore, studies of nucleic acids in these cell types are preferably performed by 1D ^1^H or ^19^F NMR (see below).

Isotopic labeling strategies are generally required to filter the ^1^H cellular background signals. However, exceptions can be found when signals from the macromolecule of interest fall in regions of the cellular ^1^H NMR spectrum that are mostly background-free. These regions, regardless of the type of cell analyzed, include negative ^1^H parts per million (ppm) values, typical of side-chain methyl groups negatively shifted by ring currents in protein hydrophobic cores, and the so-called imino region between ∼11 and ∼16 ^1^H ppm, typical of imino protons of nucleic acids involved in hydrogen bonds. This in spite of the abundance of folded proteins and nucleic acids present in a cell, likely because single macromolecules are present at a very low concentration, and their tumbling rate is often too slow to give rise to observable ^1^H signals. The negative ppm region can be exploited to quantify the relative amount of a folded protein or to qualitatively assess the presence and tumbling rate of the protein of interest.^88,90^ The imino region has found broad application in the study of nucleic acids delivered to oocytes or human cells. Indeed, given the high cost of the reagents required to synthesize isotopically labeled DNAs and RNAs in the large amounts required for cellular delivery, being able to observe unlabeled nucleic acids in the background-free ^1^H imino region represents a useful compromise.^102,105,112^ Signals arising from nitrogen-bound protons in the imidazole ring of metal-coordinating histidines can also fall in the imino region of the ^1^H spectrum. Usually, these moieties are not observable in the ^1^H spectrum, as they are broadened due to solvent exchange. However, metal-coordinating histidines in metalloproteins are often involved in strong hydrogen-bonding networks, thus making these signals readily detectable in the spectrum. This feature has been extensively exploited by the Banci group to investigate superoxide dismutase 1 and carbonic anhydrases without resorting to isotopic labeling.^84,87,92^

^19^F has also been employed to investigate macromolecules in living cells. Thanks to its high gyromagnetic ratio, the ^19^F isotope is highly sensitive, and it is 100% naturally abundant. Although fluorine atoms, unlike ^13^C and ^15^N, chemically alter the investigated macromolecules, with possible consequences to the conformation, stability, and activity, they are generally well-tolerated by proteins when introduced on single aromatic residues.^113^ As fluorine is not naturally present in biological systems, in-cell ^19^F NMR spectra are virtually background-free. This advantage, coupled to the small number of unique fluorine atoms present in the sample, makes it possible for one to analyze cell samples by simple 1D NMR experiments without incurring a spectral overlap. ^19^F labeling was first applied to observe proteins in bacteria by Mehl and coauthors, who employed a modified aminoacyl-tRNA synthetase to insert trifluoromethyl-l-phenylalanine (tfmF) at specific positions along the protein sequence using the UAG stop codon.^114^ Li and Pielak then showed that labeling proteins in bacteria with 3-fluoro-tyrosine (3FY) led to a relaxation broadening of the ^19^F resonances for large or interacting proteins, whereas tfmF allowed larger proteins to be observed thanks to the more favorable relaxation properties of the fluoromethyl group.^115^ To incorporate 3FY, the free amino acid was provided in minimal medium together with glyphosate to inhibit the endogenous synthesis of tyrosine, while tfmF was introduced as previously reported.^114^ The Crowley group showed that, in bacteria, proteins could be labeled with 5-fluorotryptophan (5FW) by using the inexpensive precursor 5-fluoroindole.^116^ Because of the high efficiency and low costs involved, 5FW labeling was later employed by several groups for ^19^F in-cell NMR studies of GB1-based artificial constructs.^117−119^

The Li and Pielak groups also showed that fluorinated proteins produced in bacteria can be delivered to *X. laevis* and *D. rerio* oocytes.^15,118−120^ In principle, a CPP-mediated delivery to mammalian cells is possible, although the only known attempt by the Pielak group resulted in the interaction between the protein, 3FY-labeled CPP-αSyn, and the plasma membrane of CHO-K1 cells. The low delivery efficiency prevented an intracellular protein detection by ^19^F NMR.^121^

Finally, Xu and Srivatsan showed that ^19^F NMR can also be applied to study the conformation of nucleic acids in intact *X. laevis* oocytes, thanks to the development of fluorinated nucleoside analogues for an in vitro synthesis of ^19^F-tagged DNA and RNA, which are then injected in the oocytes for a ^19^F in-cell NMR analysis.^101,122,123^ Recently, Bao and Xu successfully observed by ^19^F NMR a fluorinated telomeric DNA–RNA-hybrid G-quadruplex delivered to human cells using the pore-forming toxin SLO approach.^124^

### 2.3 NMR Methods

Modern NMR spectroscopists can choose between a plethora of methods that are constantly developed and improved upon since the birth of Fourier transform NMR. Among these methods, heteronuclear multidimensional experiments are nowadays the most exploited in the characterization of biological macromolecules, because they can simultaneously probe different biologically relevant NMR-active, spin-1/2 nuclei, typically ^1^H, ^13^C, and ^15^N, obtain information on correlations between them—either through-bond or through-space, and can be used to monitor conformational changes at the single-residue level by a chemical shift perturbation (CSP) analysis. Combined together, these experiments allow one to perform resonance assignments, to determine de novo structures—either by classical NOE-based approaches or by exploiting paramagnetic effects, to probe the dynamic behavior of macromolecules in a wide range of time scales—from picoseconds to milliseconds, and to structurally investigate chemical modifications and intermolecular interactions.

In principle, the experiments suitable for an in-cell NMR analysis do not differ much from those normally used in vitro. However, in practice, the choice of useful experiments is limited by several factors. One of the main drawbacks of studying molecules inside cells is the reduced number of individual molecules in the sample, which is limited by the maximum concentration of the molecule under study reachable in the cells, further multiplied by a dilution factor arising from the fact that cells occupy only a fraction of the NMR tube volume. Another major issue is the lifetime of the cell sample, which depends on the type of cells employed but which generally limits the acquisition of NMR experiments to a few hours, unless bioreactor systems are employed (see section 4). Further limitations in the choice of the most effective experiments are imposed by the cellular environment, due to its effects on the relaxation properties of the nuclear spins of the observed molecules.

In light of the above limitations, in-cell NMR spectroscopy benefits from high magnetic fields and from experiments optimized for maximum sensitivity, that is, those providing the highest signal-to-noise ratio per unit of time. Higher magnetic field strengths (*B*_0_) increase the nuclear spin energy splitting and provide a gain of sensitivity proportional to *B*_0_^3/2^. Although higher fields adversely affect the spin relaxation when studying slow-tumbling molecules, transverse relaxation optimized spectroscopy (TROSY)-type experiments can overcome this limitation, making ultrahigh fields appealing for high-resolution and high-sensitivity in-cell NMR.^125^

Of the many types of NMR experiments suitable for biomolecules, 2D, ^1^H–^15^N/^1^H–^13^C correlation spectra—such as heteronuclear single- or multiple-quantum correlation (HSQC or HMQC, respectively)—are among the most used, as they quickly provide a fingerprint-like snapshot of the conformation of macromolecules, are excellent “starting points” for resonance assignment and structure determination, and can be used for a CSP analysis. In the first works describing protein in-cell NMR in *E. coli*, conventional ^15^N and ^13^C HSQC spectra were recorded, which were the state-of-the-art at the time.^60,107^ Since then, several variations of these NMR spectra have been developed, including the so-called fast-pulsing NMR methods, which manipulate differently the magnetization of the observed and nonobserved nuclei in order to greatly reduce the longitudinal relaxation time of the observed nuclei and therefore allow shorter interscan delays with minor signal loss, greatly improving the sensitivity. Among these, the SOFAST-HMQC and BEST-type experiments have found widespread use,^126,127^ and more recently ALSOFAST-type experiments have further improved the performance of ^1^H–^13^C methyl correlation spectra.^128,129^ For ^1^H–^15^N correlation spectra, the SOFAST-HMQC pulse sequence is likely the most commonly used, due to its simplicity and high sensitivity in various sample conditions for both folded^17,84^ and unfolded proteins^39^ and for nucleic acids.^102^ Compared to folded proteins, intrinsically disordered proteins have a much lower amide ^1^H chemical shift dispersion, which may pose additional challenges for an in-cell analysis. Heteronuclear ^13^C detection has been shown to be beneficial for improving the spectral resolution of IDPs, thanks to the improved signal dispersion and absence of solvent-exchange broadening.^130^ Indeed, direct ^13^C-detected NMR experiments have been shown to be useful for analyzing IDPs in cells.^108,131,132^

In order to map chemical shift changes onto the amino acid sequence of a protein, the spectral resonances must be assigned to the corresponding residues. The resonance assignment of the backbone is often sufficient for a CSP analysis. To perform the assignment, multiple sequential through-bond correlations need to be established between the nuclei of the backbone, making it necessary to record NMR experiments with three or more dimensions. A complete assignment then serves as a starting point to calculate the 3D structure of a protein, which requires 2D and often 3D nuclear Overhauser effect spectroscopy (NOESY)-type experiments. In living cells, the time limitation imposed by the short lifetime of the sample makes conventional 3D experiments requiring long acquisition times unfeasible. This limitation can be overcome by exploiting sparse sampling schemes combined with advanced processing algorithms that, unlike Fourier transform, do not need the complete time-domain coverage of the indirect dimensions.^133,134^ In the work reporting the first structure obtained by in-cell NMR, Shirakawa, Güntert, and Ito applied a sparse sampling scheme to all 3D in-cell spectra in order to reduce the total acquisition time,^56^ using a maximum entropy (MaxEnt) approach for data processing.^135^ Later, the same authors used an improved method, quantitative maximum entropy (QME), to reconstruct sparsely sampled 3D spectra for an in-cell protein structure calculation.^72,73,136^

As an alternative to an NOE-based protein structure calculation, Häussinger/Selenko and Su independently demonstrated that structural models of proteins chemically modified with lanthanide-binding tags can be obtained from in-cell paramagnetic NMR data recording only 2D ^1^H–^15^N HSQC-type NMR spectra.^137,138^ Specifically, different sets of backbone ^1^H/^15^N pseudocontact shifts (PCSs) and residual dipolar couplings (RDCs) induced by lanthanide ions with different magnetic susceptibility anisotropies^139,140^ provided a way to reconstruct the protein spatial arrangement with the aid of a structure calculation algorithm, GPS-Rosetta.^141^ In the case of IDPs, a lanthanide-binding tag conjugated at different positions along the sequence allows one to investigate protein dynamics in-cell by measuring the intramolecular paramagnetic relaxation enhancement (PRE) effect, which decreases as a function of the average distance from the paramagnetic center and can report on the existence of compact states.^39^

Protein backbone dynamics can be studied directly in living cells by recording 2D heteronuclear relaxation experiments. Examples of ^15^N *T*_1_, *T*_2_, and NOEs measured by recording 2D spectra on uniformly ^15^N-labeled proteins have been reported both in bacteria and in human cells.^39,142^ Recording these spectra can be time-consuming, due to the long recycle delays required, making them unsuitable for short-lived cell samples. Alternatively, 1D ^15^N-edited ^1^H spectra can be used to measure protein ^15^N *T*_1_ and *T*_2_ by employing an amino acid type-selective labeling scheme to reduce the crowding in the 1D spectra.^56,143^

Solvent exchange rates of backbone amides can be measured by NMR over a broad range of time scales, and they are useful for investigating the thermodynamics of protein folding at a single residue resolution. The rate of slow-exchanging amides can be obtained by time-resolved 2D NMR, either directly by hydrogen–deuterium (H-D) exchange in living cells^17^ or indirectly by measuring the H-D exchange of quenched cell lysates, as described by the Pielak group.^144^ The rate of fast-exchanging amides, typical of IDPs, can be measured in intact cells with pseudo-3D HSQC-type spectra where the rate of exchange is encoded in the pseudodimension, such as the CLEANEX-PM,^145^ which does not rely on H-D exchange,^39,146^ or the SOLEXSY experiment,^147^ which is specifically tailored for fast-tumbling amides and was applied on bacteria suspended in 50%/50% H_2_O/D_2_O buffer.^148^ D_2_O has minimal effects on bacterial cell viability over the duration of the above experiments.^144^

The translational diffusion of a molecule can be greatly affected by the cellular environment, depending on the viscosity and on the strength of interactions with other macromolecules. Traditionally, diffusion-ordered spectroscopy (DOSY) and similar methods are applied in vitro to separate signals from different molecules in complex mixtures.^149^ These experiments rely on pulsed field gradients to encode the position of a spin along the *z*-axis before the evolution, which is then refocused with an opposite gradient. The diffusion of molecules leads to a decrease in the amount of signal that is refocused after the evolution, and signals from fast-diffusing molecules will decrease more steeply than slow-diffusing ones, as a function of the evolution time. The slow diffusion of macromolecules such as proteins requires tailored NMR experiments, such as those based on the heteronuclear stimulated-echo (X-STE), which exploit the long *T*_1_ of heteronuclear spins to preserve the magnetization during longer evolution times.^150,151^ In cells, where translational diffusion is further reduced, the approach has been pushed to its limits by Dobson and Christodoulou, who showed that ^15^N- or ^13^C-edited diffusion experiments allowed one to study the diffusion behavior of proteins in bacteria, even in the presence of interactions with cellular components, and could be employed as effective filters to distinguish intracellular from extracellular proteins.^152^

In addition to the above approaches relying on ^13^C and ^15^N, ^19^F NMR has also been applied to probe the conformational dynamics and diffusion of macromolecules in living cells. ^19^F *T*_1_ and *T*_2_, measured by inversion recovery and Carr-Purcell-Meiboom-Gill (CPMG), respectively, allow probing the nanosecond time-scale motions of a fluorinated protein both in the folded and in the unfolded state, as shown in bacterial cells by Pielak and Li.^153−155^ 2D ^19^F exchange spectroscopy (EXSY) has been applied by Shirakawa and Hamachi to measure the exchange kinetics in-cell between free and bound conformations of a fluorinated probe conjugated to a globular protein.^156^

The resolution and sensitivity of solution NMR spectroscopy is heavily dependent on the rotational diffusion of the observed molecules. Nuclear spins of slow-tumbling macromolecules, such as folded proteins or nucleic acids, experience faster transverse relaxation rates (*R*_2_) compared to those of small molecules, leading to broader and lower signals in the NMR spectra. This effect increases as a function of molecular size, making solution NMR impractical above certain molecular sizes. In the cellular environment, the tumbling of a molecule further decreases as a function of its interactions with large cellular components. TROSY-based NMR experiments allow one to detect signals from slower-tumbling molecules, by selecting spin states that relax more slowly thanks to cross-correlated transverse relaxation mechanisms.^157^^1^H–^15^N TROSY spectra have been applied to bacterial cells to probe the extent of transient interactions in bacteria,^158^ and they have proven useful for in-cell NMR at ultrahigh fields,^125^ where the field dependence of the HN-TROSY enhancement reaches a maximum.^159^ Aromatic ^1^H–^13^C TROSY can be applied to investigate H–C correlations in ^13^C-labeled nucleic acids in oocytes.^102^ When protein side-chain ^13^CH_3_ groups are selectively labeled, the standard ^1^H–^13^C HMQC pulse sequence already benefits from a TROSY enhancement.^160^ This approach, termed methyl-TROSY, results in an increased resolution and sensitivity when slow-tumbling macromolecules are analyzed. A deuterated background is required to properly decrease the *R*_2_ in slow-tumbling molecules,^161^ as shown by the Shimada group.^37^ In the case of molecules involved in high-molecular weight complexes (greater than ∼100 kDa), the ^1^H transverse relaxation is so fast that it leads to an excessive loss of magnetization during the pulse sequence itself, preventing signal detection. Cross-relaxation enhanced polarization transfer (CRINEPT)-based experiments partially overcome this loss, by shortening the time required to transfer the magnetization from ^1^H to ^15^N and back, and allow one to detect a heteronuclear NMR analysis of complexes up to ∼1 MDa size.^162^ The Shekhtman group showed that CRINEPT-based experiments coupled with protein deuteration allowed the observation of proteins, otherwise “invisible” due to interactions with the ribosome, both in bacteria and human cells.^163,164^

In addition to the homogeneous line broadening caused by the aforementioned transverse relaxation mechanisms, in-cell NMR spectra also experience inhomogeneous line broadening caused by micrometer-scale changes in the magnetic susceptibility of the different cellular compartments/membranes. Further field inhomogeneities could be introduced by nonideal sample geometries. Given the low signal-to-noise ratio of the signals of interest, the analysis of in-cell spectra can be challenging regardless of the choice of NMR experiment, due to artifacts arising from poor water suppression, strong background signals, severe overlap caused by high spectral complexity, and inhomogeneous line broadening. Relevant information hidden in the crowded, noisy in-cell NMR spectra can be extracted by deconvolution or decomposition methods.

Signal deconvolution can be applied to resolve signals in overlapped 1D in-cell spectra and is typically employed in a ^19^F line-shape analysis to retrieve the line width of signals as a function of temperature or interactions.^118,120^ The deconvolution of overlapped signals can also be employed to retrieve signal intensities in regions of the ^1^H 1D spectrum free from cellular background signals.^92^ Signal deconvolution also contributes to reducing the impact of inhomogeneous line broadening in 2D spectra, with the aim of improving the measurement accuracy of backbone chemical shifts for the secondary structure prediction.^165^

Decomposition methods based on linear algebra have also proven useful to extract the relevant components in a series of complex in-cell NMR spectra. The Shekhtman group showed that a singular-value decomposition (SVD) analysis can extract meaningful changes in signal intensity in a series of spectra from cell samples expressing different levels of interacting proteins^166,167^ and to screen for inhibitors of intracellular protein–protein interactions.^168^ The analysis of real-time in-cell NMR spectra greatly benefits from spectral decomposition approaches. The Shekhtman group applied SVD to identify meaningful time-dependent changes in real-time series of spectra.^164,169^ Later, Luchinat and Banci applied an iterative algorithm, multivariate curve resolution by alternating least-square fitting (MCR-ALS),^170^ to reconstruct the pure spectra and the concentration profiles of free and ligand-bound intracellular protein fractions as a function of time from real-time series of 1D and 2D NMR spectra.^110,171,172^

## 3 Applications

### 3.1 Crowding and Folding Stability

The interior of a cell is a quite crowded environment where the macromolecule concentration can reach values over 300 g/L.^173^ Such a complex intracellular space affects not only the protein function and structure but also the folding thermodynamics. In-cell NMR is arguably the best approach to investigate how crowding affects protein folding, as it allows one to characterize proteins at the residue level in the intact cell environment. Classically, the excluded volume effect caused by macromolecular crowding has been considered as the main factor determining the differences in protein folding free energy between the cellular environment and a diluted solution.^174^

The Pielak group first highlighted that nonspecific interactions and, in particular, the electrostatic ones may have a strong influence on the thermodynamic stability of intracellular proteins,^175^ and the group extensively investigated protein folding in the bacterial cytoplasm in a series of seminal works. The characterization of the folding state of the mutated and marginally stable streptococcal immunoglobulin G binding domain of protein L (ProtL) showed that, contrary to predictions, the excluded volume effect of the *E. coli* cytoplasm was not sufficient to stabilize the folded state of the protein, indicating that destabilizing nonspecific interactions overcame the effect of volume exclusion.^176^ It was further observed that weak, attractive interactions occurring in the crowded environment can largely affect the thermodynamics of the folding process, as reported for the B1 domain of protein G (GB1). Through the measurement of GB1 hydrogen–deuterium (H–D) exchange rates in vitro and in-cell the free energies of opening, Δ*G*°′_op_ (regarded as equivalent to local unfolding free energies) was determined for each residue.^144^ Overall, GB1 was found to be less stable in cells than in a diluted solution.^177^ Furthermore, small changes in the surface properties of the protein can dramatically change the effect of the cytoplasm. A single mutation on the GB1 surface was found to destabilize the protein ∼10-fold more in cells than in vitro, and the energies involved in the cytoplasm–GB1 interactions were estimated to be as large as those typically observed in specific protein–protein complexes.^178^^19^F NMR was exploited for investigating the folding-unfolding equilibrium of the SH3 domain of the *Drosophila* signal transduction protein, revealing that charge–charge interactions involving protein surfaces have a fundamental role in determining the cytoplasmic effect on the thermodynamics and kinetics of folding.^154^ Notably, in addition to modulating the stability of the folded state, the intracellular environment was also found to affect the unfolded state of a protein.^179^

Molecular crowding effects are strictly correlated with the so-called “quinary structure”. This term was first coined in 1973 by Vaĭnshteĭn^180^ to indicate the fifth-order level of organization of macromolecules and was subsequently used by Edelstein and McConkey, who postulated the biological relevance of a high-order intracellular organization made by weak, transient interactions.^181,182^ In light of their recent NMR observations, Cohen and Pielak redefined the quinary structure as the ensemble of all transient interactions that not only contribute to the structure of macromolecular complexes but also influence the overall organization in the intracellular environment.^179^ Among them, electrostatic interactions have a dominant role. Indeed, monitoring the interactions of the folded/unfolded state of GB1 as a function of pH within the *E. coli* milieu, it was observed that, at low pH (5.0), most of the *E. coli* proteins take the status of polycations, thus interacting with the more accessible surfaces of the unfolded forms of GB1 and contributing to destabilize the protein.^183^ A comparable destabilization effect was observed also in the presence of hyperosmotic shock.^184^ The reduction of the intracellular volume due to the efflux of water resulted in overcrowding inside the cell and consequently in an increase of transient attractive interactions that destabilized the SH3 protein. Protein stability in human cells was investigated by the Oliveberg group. The SOD1 β-barrel (i.e., SOD1^ΔIVΔVII^) was used as a model system, as it shows a simple two-states folding equilibrium.^29,46^ By comparing the temperature dependence of the folding free energy in vitro, in *E. coli* and in A2780 cells, it was found that the weak interactions of the SOD1 β-barrel with the cellular components led to the stabilization of the unfolded state, with a consequent decrease of the melting temperature. Notably, the human cytoplasm was found to be more destabilizing at higher temperatures, whereas the bacterial one had a stronger effect at low temperatures, due to the different composition of the two environments.

**Figure 2:**
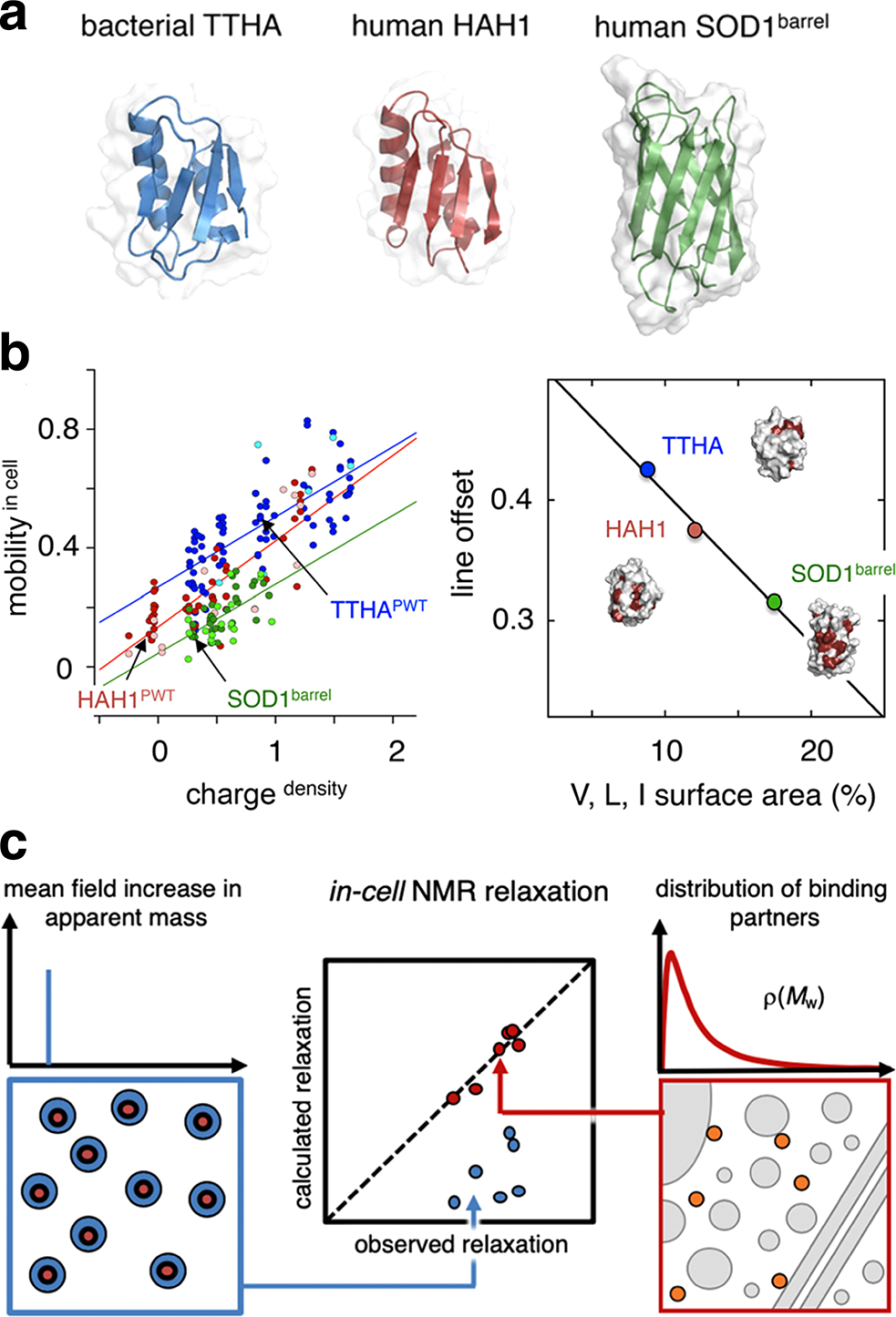
Protein quinary interactions decoded by in-cell NMR. (a) 3D structure of three evolutionary divergent proteins: bacterial TTHA, human HAH1, and human SOD1 β-barrel; (b) the rotational mobility in bacterial cells of a set of surface mutations for the proteins shown in (a) depends both on the negative charge density (left) and on the exposed surface area of the hydrophobic side chains of valine (V), leucine (L), and isoleucine (I) residues (right): increasing charge density and decreasing hydrophobic surface result in faster rotational diffusion. Mobility^in cell^ is calculated as the ratio of in-cell peak heights and lysate peak heights. Line offset in the right panel is calculated from the regression lines (left panel) at density charge^density^ = 1. Reproduced with permission from Mu et al.^185^ Copyright 2017 Mu et al. (c) Comparison of models to describe in-cell NMR relaxation data: an apparent mass increase of the observed protein (left) results in poor agreement between *R*_1_ and *R*_2_, whereas a model where the observed protein is transiently interacting with cellular components of different sizes (right) fully reconciles the *R*_1_ and *R*_2_ data. Reproduced with permission from Leeb et al.^143^ Copyright 2020 American Chemical Society.

As mentioned above, protein surfaces strongly determine quinary interactions. The Oliveberg group used in-cell NMR to investigate how surface mutations could influence protein rotational diffusion in bacteria.^185^ The motions of three different proteins (bacterial TTHA, human HAH1, and SOD1 β-barrel) were compared with those of a series of respective charge mutants (Figure 2a). While the three proteins have different rotational diffusion rates in the intracellular environment, due to different sizes and intrinsic behavior, a comparison between each wild-type protein and its charge mutants revealed a consistent dependence on physicochemical parameters like net charge density, surface hydrophobicity, and electric dipole moment (Figure 2b). The same group recently reported that the intracellular heteronuclear longitudinal and transverse relaxation rates of the same set of proteins and charge mutant series could not be interpreted with a simplistic “increased viscosity” model.^143^ Instead, the relaxation rates were consistent with the occurrence of fast-exchange equilibria between free protein and protein bound to high-molecular weight species (Figure 2c). The transient interactions were also quantified for each protein and mutant series, finding a relationship between the charge of the proteins and the estimated bound fractions, where proteins with a less negative net charge are more prone to establish interactions with high-molecular weight cellular components.

In extreme cases, transient interactions with large components of the cellular environment may cause NMR signal broadening beyond detection. Unfortunately, such extreme cases are not uncommon when macromolecules are studied by solution in-cell NMR. The rotational diffusion properties of proteins inside living cells have therefore been the object of many in-cell NMR studies. By measuring ^1^H relaxation in perfused myocardium and intact erythrocytes, it was found that the rotational correlation times of myoglobin and hemoglobin were, respectively, ∼1.4 and ∼2.2 times longer inside the cells than in diluted solution.^186^ In a similar work, ^19^F NMR was employed to study the behavior of three glycolytic enzymes, namely, hexokinase, phosphoglycerate kinase, and pyruvate kinase, in *S. cerevisiae*, again reporting a viscosity approximately twofold higher than that of water.^187^ Later, the Gierasch and Li groups observed that, in bacteria, the viscosity of the cellular environment is somewhat higher, three- to eightfold higher than that of water, but still too low to explain the increased transverse relaxation observed for some proteins.^153,158^ In *X. laevis* oocytes, the Li group reported a viscosity ∼1.2 times higher than that of water.^188^ Furthermore, the same group measured ^15^N and ^19^F relaxation rates on concatenated GB1 constructs of increasing molecular weight, in bacteria and oocytes, and found that the transverse relaxation did not depend on the viscosity alone and had a different size dependence, likely due to the different molecular composition of the two environments.^155^ Therefore, the above results indicate that the weak, transient interactions with the cellular environment, the same interactions that can affect protein folding thermodynamics (see above), are mainly responsible for the slow rotational diffusion of proteins.

In addition to the weak, diffuse interactions, proteins can also experience stronger interactions with certain cellular components. The Crowley group studied the origin of these strong interactions and their effect on the rotational diffusion of cytochrome c (Cyt c) expressed in *E. coli*. Not only did the Cyt c prove to be completely NMR-invisible in intact cells but it was also undetectable in the cell lysate, indicating that it experiences interactions with high-molecular weight components that were strong enough to persist upon dilution of the cellular content.^189^ Whole-cell lysate size-exclusion chromatography (SEC) at increasing salt concentrations indicated that such interactions were mainly electrostatic in nature and occurred between negatively charged molecules and the positively charged surface of Cyt c. The same group investigated the charge–charge interactions occurring between the Lys/Arg-rich HIV-1 Tat peptide fused to GB1 and intracellular molecules, which prevented NMR detection,^190^ and later found that increases in the length of a poly-Arg tail attached to the GB1 protein determined the increase of electrostatic interactions with negatively charged components, causing line broadening beyond detection.^117^ Our group investigated the extent and the nature of functional interactions versus diffuse, nonspecific interactions experienced by profilin 1 (PFN1), a human protein required for actin polymerization and interacting with many other functional partners.^90^ In addition to the finding that PFN1 experienced different types of interactions in bacteria and human cells (see section 3.2), it was observed that some interactions with bacterial components were strong enough to still be present even after cell lysis. Notably, while the works described above did not explore in detail the molecular nature of the strongly interacting, nonspecific partners causing NMR line broadening, some insight came from further processing the cell lysates. Indeed, a treatment with ribonuclease A in the presence of Mg^2+^ disrupted the residual interactions experienced by both Tat-GB1 and PFN1 in bacterial cell lysates, leading to sharper and stronger NMR signals,^90,190^ thus suggesting that, for those proteins, the line broadening was caused by the interaction with bacterial RNA.

Different growth conditions alter the cellular metabolic pathways, which can also affect the localization and interactions of the intracellular proteins. In the yeast *P. pastoris*, changing the carbon source resulted in different cellular localizations and the rotational diffusion of overexpressed ubiquitin.^64^ With methanol as a carbon source, ubiquitin was diffusely localized in the cytosol and NMR-visible, while in a mixed dextrose/methanol medium it was mainly localized in intracellular vesicles and undetectable by NMR. The finding that the metabolic cellular state strongly influences the quinary structure of ubiquitin later prompted the Shekhtman group to identify a connection between metabolic state, cellular RNA content, and quinary interactions. The group estimated the average size of molecular complexes formed between the globular proteins thioredoxin, FKBP, adenylate kinase (ADK), and ubiquitin and intracellular molecules in bacteria and Hela cells by optimizing the transfer delay in CRINEPT-TROSY spectra.^47^ The same proteins were observed in vitro in the presence or absence of RNA, confirming that RNA was mainly responsible for the quinary interactions in cells. The same group further investigated the ubiquitin–RNA interaction in yeast.^65^ Immunofluorescence microscopy analyses revealed that the colocalization of RNA and ubiquitin increased under a dextrose/methanol carbon source. Notably, the metabolism switch dramatically altered the total RNA content, further confirming the role of RNA on the ubiquitin quinary interactions. The interaction between proteins and ribosomal particles was also examined. By combining in-cell/in vitro NMR with other biophysical assays it was found that ADK, dihydrofolate reductase, and thymidylate synthase bind ribosomes with a micromolar affinity and that such an interaction modulates their enzymatic activity.^163^ The rotational diffusion of green fluorescent protein (GFP) was also affected by ribosome binding. Overall, these findings led to the hypothesis that the quinary structures mediated by ribosomes and RNA molecules are fundamental in the suppression and activation of specific protein functions.

### 3.2 Protein–Protein Interactions

Interactions between macromolecules are at the basis of the mechanisms that exert and regulate most of the functional processes within the cell. To exert their biological activity or to complete their maturation process, many proteins need to establish specific interactions with one or more macromolecular partners. NMR spectroscopy has proven to be one of the most powerful techniques for investigating these interactions, as it allows characterizing the interactions in solution under physiological conditions, and is ideally applied to study weak, transient interactions. The atomic resolution provided by NMR allows one to examine in detail the residues of each protein partner involved in the interaction, thus providing structural information on the complex and contributing to the elucidation of its molecular mechanisms. The most common and informative approach for studying protein–protein interactions by NMR is the CSP analysis. Indeed, the interaction surface and the binding affinity can easily be determined by the analyses of the chemical shift changes that occurred after the formation of a protein–protein complex.^191^ The development of in-cell NMR spectroscopy approaches has opened new opportunities to investigate protein–protein interactions directly in the physiological context of the cellular environment.

When directly expressing isotope-labeled proteins in the cell, a simultaneous expression results in the undesired labeling of both partners, which complicates the NMR analysis. The Shekhtman group developed the STructural-INTeractions using NMR spectroscopy (STINT-NMR) approach to evaluate protein structural changes upon interaction with a partner in bacteria.^192,193^ The approach is based on the time-controlled sequential expression of two or more proteins. The target protein overexpression is induced in *E. coli* cells in the presence of labeled medium, and then the partner protein expression is induced after a switch to a nonlabeled medium. This makes sure that only one protein is labeled, thus decreasing the overlap in the NMR spectra and allowing one to correctly interpret the spectra. STINT-NMR was applied to monitor the interaction between ubiquitin and partners with different affinities and molecular weight: a 28 aa peptide derived from ataxin 3 protein (AUIM) and two other proteins, signal transducing adaptor molecule (STAM2), and the hepatocyte growth factor regulated substrate (Hrs), belonging to the tyrosine kinase receptor endocytic sorting machinery.^55,194^ The protein–protein interactions were revealed by changes in 2D correlation spectra, which allowed one to identify the binding interfaces by a CSP analysis. By expressing the Src-family tyrosine kinase Fyn, the authors induced the phosphorylation of STAM2 and Hrs and observed that the number of perturbed residues of ubiquitin upon interaction with the phosphorylated partners changed with respect to the interaction with nonphosphorylated partners. Furthermore, the phosphorylation state of two STAM2 tyrosines modulated the interaction surface of the ubiquitin-STAM2-Hrs ternary complex.^194^

The interaction of ubiquitin-like protein Pup with the mycobacterium proteosome ATPase (Mpa) and with the entire proteasome complex that comprises Mpa and the mycobacterial proteasome core particle (CP) was also investigated through STINT-NMR by the same group.^195^ Pup-GGQ is a highly dynamic unstructured protein that is responsible for tagging proteins for a proteasomal degradation. The in-cell NMR analyses showed that Pup-GGQ N- and C-terminus residues weakly interact with the proteasomal ATPase Mpa. However, when the entire proteasome complex was overexpressed, a larger number of Pup-GGQ residues became involved in the binding, thus demonstrating a strong interaction.

The conventional analyses focused on determining the interacting residues often do not take into account the signals arising from cellular changes during NMR acquisition time and those arising from nonspecific interactions. This may lead to an incorrect estimate of the residues involved in the specific interactions. To solve this problem, the Shekhtman group applied an SVD analysis (see section 2.3) to previously collected data.^166^ The method was able to discriminate between specific and nonspecific binding and revealed that the Pup residues, for which signal intensity changes were observed, could be divided in two classes based on their dependence on the Mpa expression levels: residues with larger singular value (SV) contributions were attributed to the specific interaction with Mpa, while those with smaller SV contributions were attributed to changes in nonspecific binding to other cellular components.

Protein–Protein interactions exert a crucial role in protein folding and maturation pathways in human cells. In-cell NMR has contributed to elucidate several functional aspects of the interaction between human superoxide dismutase 1 and its specific metallochaperone CCS (see section 3.4).^84,86,87^ While the effects of the SOD1 interaction with full-length, active CCS on the metalation and redox state of SOD1 were clearly observed, the SOD1-CCS complex escaped NMR detection, due to the intrinsically transient nature of the chaperone-client complex and to the fact that full-length CCS is undetectable by in-cell NMR due to diffuse interactions.^84,87^ Instead, the stable heterodimeric complex formed between immature SOD1 and the SOD-like domain 2 of CCS (D2), responsible for the interaction with SOD1, was successfully observed.^86^ The complex gave rise to clear signals in the in-cell NMR spectra, but the strong spectral overlap caused by the identical ^15^N-labeling of the two partners prevented a detailed analysis.

For the above purpose, a method for selectively labeling only one of two partners expressed in mammalian cells, similar to the STINT-NMR approach in bacteria, would be required. Luchinat and coauthors achieved the selective labeling of one of two partners in human cells relying on the combination between a constitutive and transient protein expression with gene silencing (see section 2.1.2).^94^ The system feasibility was assessed by sequentially expressing the copper binding HAH1 and SOD1 proteins. Although this approach has not been applied further, it may reveal a useful method for studying protein–protein interactions.

Intracellular proteins can experience weak, diffuse interactions with the crowded cellular environment, causing a decrease in the rotational diffusion and leading to signal broadening beyond detection. As described in the previous section, studies performed by in-cell NMR have provided insights on the nature of these interactions and on their consequences on protein function (see section 3.1). In addition to nonspecific interactions, intracellular protein partners can specifically interact with the protein of interest. The occurrence of such specific interactions in eukaryotic cells was shown by the Shirakawa group to be responsible for the signal broadening beyond detection of yeast ubiquitin delivered into *X. laevis* oocytes.^9^ Introducing specific mutations on the hydrophobic residues Leu8, Ile44, and Val70, known to be involved in the interaction between ubiquitin and its partners, gave rise to well-resolved signals in the spectra, thus implying that the signal broadening observed with the wild-type ubiquitin was caused by the interaction with specific proteins. The same line broadening caused by the specific intracellular partner interaction was later observed when ubiquitin was delivered into HeLa cells by using cell-penetrating peptides.^17^ However, in general, the occurrence of multiple types of interactions, both specific and nonspecific, cannot be ruled out.

The coexistence of both kinds of interactions was demonstrated by the Dötsch group on the peptidyl-prolyl isomerase Pin1 in *X. laevis* oocytes and extracts.^196^ In that work, the intracellular interactions of the N-terminal Trp-Trp binding module (WW) domain of Pin1, responsible for the signal broadening beyond detection, were completely abolished upon phosphorylation of Ser16 in the active site of WW by protein kinase A. The phosphomimic S16E mutant showed the same behavior, suggesting the occurrence of nonspecific interactions that were abolished by increasing the negative net charge. Notably, however, the binding of a peptide to the active site as well as the active-site mutant W34A also abolished the interactions with the environment, suggesting that interactions with intracellular target proteins are also present.

In order to discriminate between specific and nonspecific interactions, our group focused on the human profilin 1 (PFN1), a small cytosolic protein that interacts with many different functional partners in the human cell, namely, G-actin monomers, phosphatidylinositol (4,5)-bisphosphate [PtdIns(4,5)P_2_], and proteins containing poly-l-proline (PLP) motifs. These functional partners, which interact with distinct surface regions of PFN1, are absent in bacteria. Therefore, comparing the rotational mobility of PFN1 in bacteria and human cells could reveal the extent of functional versus nonspecific interactions.^90^ Wild-type PFN1 was not detectable by in-cell NMR, not only in human cells, as expected, but also in bacteria. Mutations of surface residues involved in the interaction with different partners were additively introduced, resulting in the gradual recovery of the in-cell NMR signals of the protein, however, with strikingly different patterns between bacteria and human cells. In bacteria, mutations on the region responsible for the interaction with [PtdIns(4,5)P_2_], which decrease the net charge of the protein, were sufficient to recover the NMR signals. Instead in human cells, where PFN1 interacts with specific partners, all three interaction surfaces had to be abolished, regardless of the protein net charge, to recover the NMR signals.

### 3.3 Protein Structure Determination

The knowledge of the structure of biomolecules has provided and is providing an essential contribution to the description and understanding of functional processes. Biomolecular structures are essentially obtained through in vitro techniques, such as X-ray crystallography, NMR (mainly in solution), and, more recently, cryogenic electron microscopy (cryo EM). However, the structures of macromolecules within intact living cells may differ from those determined in vitro due to the presence of multiple interactions occurring in a crowded environment. Currently, in-cell NMR represents the only methodology able to determine the structures of biomolecules in their native environment at an atomic resolution.

The first 3D structure to be determined de novo in living cells, that is, using data from in-cell NMR spectra only, was that of the putative heavy-metal binding protein TTHA1718 of *Thermus thermophilus*, a small globular protein that was highly overexpressed in *E. coli* cells, reaching 3–4 mM concentration.^56^ Different labeling strategies were adopted, such as ^13^C–^15^N for backbone assignment and selective incorporation of ^1^H,^13^C-methyl groups in a deuterated medium for side-chain assignment. To overcome the problem of the short lifetime of cells inside the NMR tube, a nonlinear sampling scheme was adopted to reduce the acquisition time of 3D spectra, and, in addition, several fresh samples were used for each experiment. Classical NOE-based distance restraints from 2D and 3D NOESY spectra were used for determining the protein structure. Despite the remarkable achievement, the high protein concentration required severely limits the applicability of this method. The Ito group reported an improved workflow for in-cell structure determination, which partially overcame the original limits by implementing more advanced methods, namely, quantitative maximum entropy (QME) for the processing of NMR data, FLYA algorithm-based automatic assignment procedure,^197^ and a Bayesian structure refinement. The workflow was applied to determine the structure of the *Streptococcus* G B1 domain (GB1) in *E. coli* cells at an ∼250 μM concentration.^136^ Recently, the same group reported a de novo protein structure determination in insect cells of five different proteins: rat calmodulin, human HRas, human ubiquitin, and the prokaryotic TTHA1718 and GB1.^73^ The proteins were expressed in sf9 insect cells transfected with the baculovirus system. A bioreactor system was used to preserve cell viability up to 24 h, extending the useful time window for NMR spectra acquisition. For GB1, the backbone resonances and most of those of the aliphatic side chains were unambiguously assigned from 3D triple resonance spectra, 3D ^15^N- and ^13^C-resolved NOESY spectra, and HCCH-TOCSY recorded on samples selectively labeled with ^13^C/^15^N-alanine, isoleucine, leucine, and valine. In-cell ubiquitin and TTHA1718 structures were calculated by using the structural restraints obtained from 3D NOESY spectra in cell, while the chemical shift assignments were retrieved from the in vitro data, due to the small chemical shift differences between the spectra in sf9 cells and in a diluted solution for both proteins. For the larger proteins, calmodulin and HRas, a strategy based on 2D ^1^H–^15^N HSQC and 3D ^15^N-resolved NOESY spectra resulted in a heavy cross-peak overlap. Therefore, ^1^H–^13^C HSQC and ^13^C-resolved NOESY experiments were recorded on samples with methyl- and aromatic-selective ^1^H,^13^C-labeling, which provided meaningful structural data for intracellular proteins with a molecular weight over 15 kDa. Overall, the in-cell structures were mostly superimposable with those determined in vitro. The accuracy varied slightly between proteins, and some local structural elements were not well-resolved, likely depending on a lower number of usable NOE restraints rather than on an actual increase of local dynamics with respect to the protein in vitro. The in-cell structure of GB1 was the most accurate and highlighted a slightly different relative orientation of the α-helix with respect to the β-sheet, compared to the structure in solution, possibly caused by interactions with the intracellular environment.

Overall, the above works proved that an NOE-based structural determination is a valid approach to obtain protein structures inside living cells. However, the approach remains strongly limited by the long acquisition times required and the complex and expensive labeling strategies necessary for side-chain assignment and NOE measurement. To address these limitations, the groups of Selenko and Su independently developed an approach for an in-cell structure determination based on the introduction of paramagnetic lanthanide binding tags.^137,138^ By conjugating tags loaded with different lanthanide ions to a protein, paramagnetic effects like pseudo contact shifts (PCSs) and residual dipolar coupling (RDC) can be easily and efficiently measured through simple and short 2D ^1^H–^15^N NMR experiments. PCSs depend on the distance and relative orientation between the N–H vector and the magnetic susceptivity anisotropy tensor of the lanthanide ion, while RDCs depend on the relative orientation between the N–H vector and the alignment tensor of the molecule.^139,140^ Therefore, both effects provide useful structural restraints that can be used to determine accurate structural models with the GPS-Rosetta program.^198^ Both groups focused on the protein GB1, which was first chemically modified in vitro through a site-specific conjugation of paramagnetic labels and was subsequently delivered into oocytes. In both studies, by using tags loaded with different lanthanide ions in different positions of the protein, an accurate GB1 structural model was determined, comparable with that obtained in vitro, thus demonstrating the validity of the method. The Ito group later demonstrated that lanthanide-tagged proteins could be delivered into HeLa cells and allowed one to measure in-cell PCSs and RDCs, showing that, in principle, the above approach for a protein structure determination could be extended to human cells.^49^ Therefore, the combination of the paramagnetic tagging, NMR spectroscopy, and GPS-Rosetta calculations can represent a valid tool for the characterization of protein structures inside eukaryotic cells.

### 3.4 Protein Folding and Chemical Modifications

The cellular milieu is a complex, constantly changing environment, in which organelles, macromolecules, and metabolites are synthesized and replaced continuously and cyclically. This dynamism is necessary for the survival of the cell and consequently of the whole organism. During their life cycle, proteins may need to undergo several post-translational modifications that are essential for reaching an active and mature conformation. The achievement of a biologically active final state can be accomplished in different ways, depending on the functional properties and cellular location of the protein. Membrane proteins, for example, are first synthesized in the cytosol and then, after a series of chemical modifications such as glycosylation or phosphorylation, are integrated in the phospholipidic bilayer. Even soluble proteins may require complex maturation processes to reach their mature state. Metalloproteins need to acquire cofactors like metal ions in the appropriate binding site; redox state changes of cysteine residues or disulfide bond formations are crucial for reaching a correct folding and stability. A failure to perform one or more of these steps can lead to pathological states. Indeed, protein misfolding and aggregation are implicated in the onset of irreversible and fatal neurodegenerative diseases such as amyotrophic lateral sclerosis (ALS), Alzheimer’s disease, Parkinson’s disease, Huntington’s disease, transmittable spongiform encephalopathies, and others. Therefore, the study of cellular processes by which a protein reaches its correct folding and maturation can help to elucidate some molecular, uncovered aspects of the above-mentioned diseases and to develop new possible treatments. In-cell NMR in human living cells directly expressing the proteins of interest can provide unique insights on their folding, maturation, cofactor binding, and redox-state modifications starting from the early steps of their synthesis.^199^

#### 3.4.1 Folding and Maturation

One of the proteins that has been extensively studied in recent years is superoxide dismutase 1. SOD1 is a key metalloenzyme that exerts its antioxidant role inside the cell by catalyzing the disproportionation of the superoxide anion radical, a byproduct of the respiration process, into molecular oxygen and hydrogen peroxide, thus preventing cellular toxicity caused by the superoxide anion and other derived reactive oxygen species (ROS). SOD1 binds a copper ion as a catalytic cofactor and a zinc ion, essential for the stabilization of the structure, and reaches its mature form through a dimerization. It has been shown that some mutations occurring on SOD1 are linked to familiar variants of amyotrophic lateral sclerosis (fALS) and that the toxic species is the immature, metal-free protein.^200−202^ These mutations destabilize the structure of the nascent, metal-free SOD1, preventing it from reaching the mature state and leading to misfolding and, eventually, to the formation of aggregates linked to motor neuron toxicity and death. The presence of protein aggregates rich in SOD1 has been indeed observed in the spinal cord of patients with ALS.^203,204^

**Figure 3:**
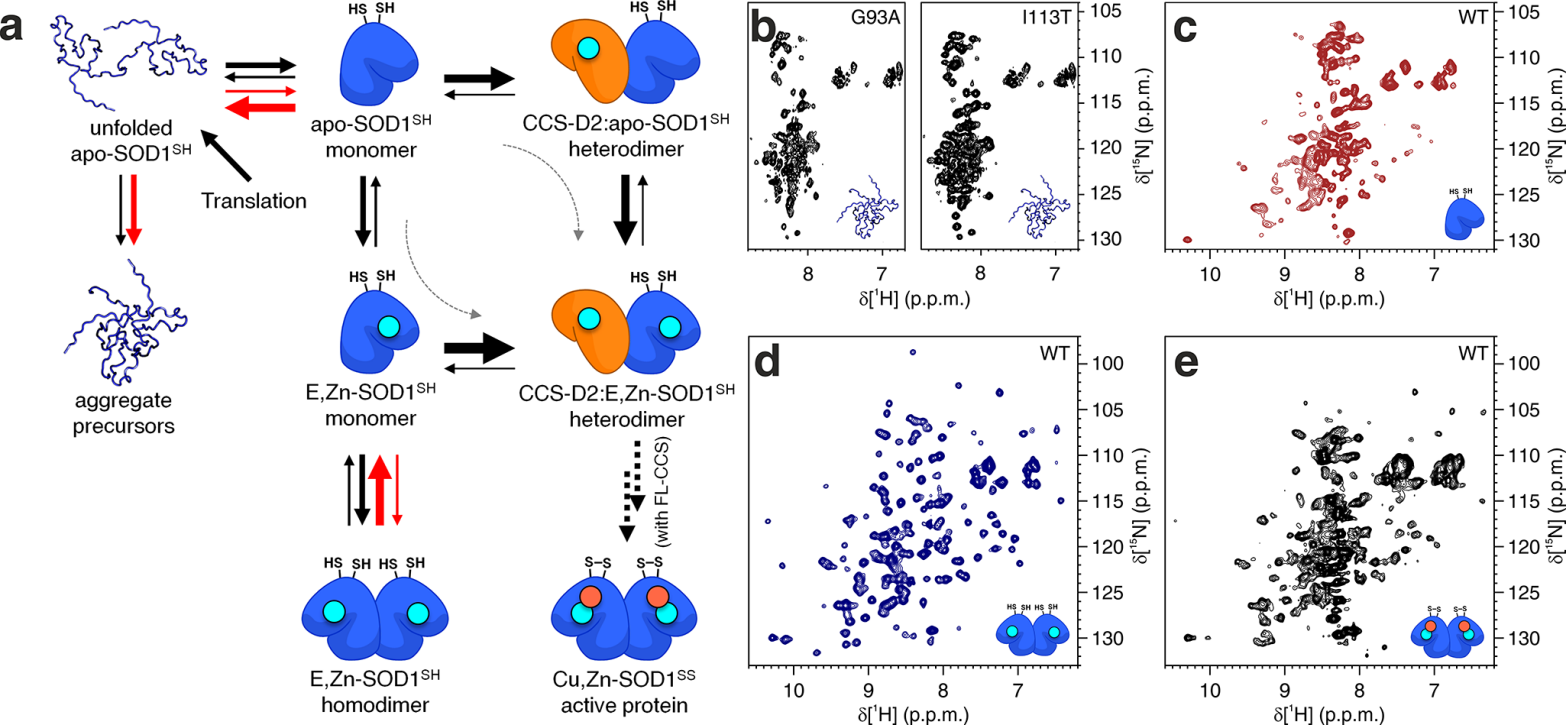
Maturation process of SOD1. (a) Scheme of SOD1 folding and maturation and molecular chaperone role of CCS. The preferred direction of each step is indicated by the size of the arrow. The effect of pathogenic mutations is shown with red arrows. Reproduced with permission from Luchinat et al.^86^ Copyright 2017 Luchinat et al. (b–e) In-cell ^1^H–^15^N NMR spectra of WT (c–e) or mutant (b) SOD1 in human cells at different steps of the maturation (indicated by the corresponding drawing): (b) irreversibly misfolded mutant SOD1. Reproduced with permission from Luchinat et al.^87^ Copyright 2014 Nature Publishing Group; (c) apo-SOD1^SH^, (d) E,Zn-SOD1^SH^, and (e) Cu,Zn-SOD^SS^ WT SOD1. Reproduced with permission from Luchinat & Banci.^84^ Copyright 2018 American Chemical Society.

In order to reach the active dimeric mature form, SOD1 must undergo several post-translational modifications including zinc binding, copper binding, and a disulfide bond formation between Cys57 and Cys146 as well as its dimerization (Figure 3a). This maturation process requires a series of concerted molecular events and involves the action of a specific metallochaperone, copper chaperone for SOD1 (CCS).^205,206^ Banci and Bertini first monitored SOD1 maturation at the molecular level by in-cell NMR, both in bacteria and human cells.^57,84^ In the latter work, SOD1 was directly overexpressed in HEK293T cells treated with different amounts of metal cofactors. With no supplementation of zinc, a large fraction of protein was found in the apo, disulfide-reduced monomeric state (apo-SOD1^SH^, Figure 3c) with a smaller fraction in the zinc-bound dimeric reduced state (E,Zn-SOD1^SH^). Once zinc was supplemented to the cells, all the protein reached the dimeric E,Zn-SOD1^SH^ form (Figure 3d). The uptake of copper has a more complex mechanism; indeed, when an excess of copper was supplemented, only a small fraction of SOD1 was fully metalated. This is consistent with the absence of free copper inside the cells: copper delivery is achieved only through specific metallochaperones for each recipient protein, such as CCS for SOD1. The coexpression of CCS with SOD1 with a supplementation of copper resulted in both copper binding and disulfide bond formation, leading to the mature form of SOD1 (Figure 3e).^84^ The in-cell NMR study of the maturation of a set of fALS-related SOD1 mutants interestingly showed that, although cells were supplemented with zinc, some SOD1 mutants are unable to bind zinc. Furthermore, the NMR spectra suggested that the metal-free mutants take a different structure with respect to that of WT apo-SOD. Indeed, the classical signals arising from the WT apo-SOD^SH^ β-barrel were not detected (Figure 3b). Quite striking was the finding that, when CCS was coexpressed together with these mutants in the presence of a copper supplementation, it was able to restore their correct maturation process that allowed zinc binding and promoted the formation of Cu,Zn-SOD1^SS^.^87^ The latter observation suggested an additional key role of CCS in the early stages of SOD1 maturation. CCS domains 1 (D1) and 2 (D2) have a globular conformation and are responsible for copper delivery and for the interaction with SOD1, respectively. Instead, domain 3 (D3) is a short and natively unstructured polypeptide segment crucial for the disulfide bond formation.^206−208^ The Banci group later showed that the coexpression of CCS D2 alone with WT SOD1 resulted in the formation of a stable complex, which, predictably, could not proceed further along the maturation pathway. Strikingly, when D2 was coexpressed together with apo-destabilizing SOD1 mutants, it led to the correct folding of mutant SOD1 and allowed zinc binding. This was shown by the detection of in-cell NMR signals of the CCS-D2/E,Zn-SOD1^SH^ heterodimer, while no unfolded state was observed, demonstrating that CCS also acts as a molecular chaperone.^86^

#### 3.4.2 Redox-State Regulation

In the cellular environment, the conformation and, consequently, the function of many proteins are strictly related to the redox state of the proteins. The formation of intra- or intermolecular disulfide bonds may be dependent on the interaction with specific partners and is essential for the correct occurrence of biological processes. As previously seen, in-cell NMR can contribute to assessing the different conformations depending on their redox state and to elucidating some aspects of their regulation. Mia40 is a mitochondrial intermembrane space (IMS) chaperone that promotes the formation of disulfide bonds on small proteins.^209,210^ Mia40 is synthesized by nuclear DNA and released in the cytoplasm in an essentially unfolded form and in the reduced state. After translocation to the IMS, thanks to specific translocators such as the translocator of the outer membrane (TOM) complex, it acquires a coiled-coil–helix-coiled-coil–helix (CHCH) fold, stabilized by two disulfide bonds.^211^ Banci and coauthors investigated the folding and redox state of Mia40 in human cells, where it was mainly localized in the cytosol, likely because its overexpression overloaded the translocation capacity of TOM channels.^88^ However, unlike what was expected at the high concentration of reduced glutathione (GSH) in the cytosol, Mia40 was found mainly in the folded and oxidized state, which is not competent for mitochondrial import. Only when glutaredoxin-1 (Grx1) or, to a lesser extent, thioredoxin (Trx) was coexpressed was Mia40 found in both reduced and oxidized states, closer to the thermodynamic equilibrium with the reduced/oxidized glutathione couple (dependent on the [GSH]^2^/[GSSG] ratio), pointing to the notion that the redox state of proteins in different compartments must be under kinetic control by specific compartment-dependent partners. The same group further investigated the redox state of SOD1, Mia40, and its substrate COX17 by in-cell NMR in cellular environments with different redox properties, HEK293T cells, *E. coli* (BL21), and *E. coli* (Origami B) strains, either with or without their redox partners.^89^ SOD1, for which the oxidized state is thermodynamically favored even under reducing conditions, was mostly present in the reduced state when in the absence of its redox partner CCS. Conversely, Mia40 and Cox17 were mostly oxidized, when in the absence of Grx1 and Trx. When the specific redox partners were coexpressed, the redox-state distribution of Mia40 and Cox17 shifted toward the expected equilibrium, whereas SOD1 was completely oxidized, thus “overshooting” with respect to the equilibrium distribution. This suggested that the redox-dependent maturation of some proteins may not equilibrate with the glutathione redox pool, as observed for SOD1, likely depending on how the redox state of the specific partner is controlled. This result is consistent with the copper-induced SOD1 oxidation mechanism proposed later, which, if confirmed, would allow H_2_O_2_ or other ROS to drive SOD1 disulfide formation regardless of the equilibrium with the glutathione pool.^208^

A more quantitative readout of the interplay between the cellular glutathione redox pool and the redox state of a protein was reported by the Shimada group.^36^ In that work, the redox state distribution of Trx and the glutathione pool were directly obtained from the in-cell NMR signals of reduced and oxidized Trx and those of GSH and GSSG, respectively, in HeLa S3 cells. By monitoring the protein response to changes in the intracellular glutathione redox potential caused by oxidative stress in a time-resolved manner, the intracellular redox curve of Trx was obtained, revealing a markedly shifted midpoint redox potential in cells (−230 mV) compared to the one measured in vitro (−300 mV). This result is consistent with the redox sensor function of Trx, which only triggers appropriate cell signaling events in the response to an actual oxidative stress, therefore, when the cellular redox potential reaches greater than −250 mV values.

Oxidative modifications of proteins are at the basis of several pathological conditions.^212,213^ alpha-Synuclein (α-syn) is an intrinsically disordered protein whose aggregation is related to Parkinson’s disease.^214,215^ It was shown that oxidative damages promote its aggregation in vitro and in-cell.^216,217^ The Selenko group applied in-cell NMR to study the oxidation-damaged α-syn in neuronal cells.^38^ α-Synuclein with all four methionines oxidized to methionine-sulfoxide was introduced by an electroporation into two different human cell lines and monitored by NMR. From the NMR experiments it was observed that only Met1 and Met5 were restored to the reduced state by the cellular methionine sulfoxide reductase repair system, while the C-terminal methionines remained oxidized. Furthermore, it was found that the methionine sulfoxides negatively affect the phosphorylation of Tyr125, impairing the overall phosphorylation pattern. These results demonstrated that oxidative stress can promote the irreversible accumulation of functionally modified α-syn inside the cells.

As seen above, in-cell NMR is a powerful approach to study protein redox-state alterations caused by external stimuli, such as oxidative stress. Indeed, it is well-known that many external factors can affect the cellular basal redox state causing increased ROS production and consequently oxidative stress. Protein deglycase DJ-1 is a dimeric protein that is correlated with some pathological states such as Parkinson’s disease, cancer, and ALS^218^ and appears to be involved in cell protection against oxidative stress.^219^ Despite being recently reported to deglycate proteins and DNA,^220,221^ the actual function of DJ-1 in the cytosol is not precisely defined.^222^ DJ-1 contains a redox-sensitive cysteine (Cys106), which in vitro can be oxidized to either sulfinic or sulfonic acid.^223^ Further evidence reported that the DJ-1 active site is able to bind both copper and zinc in vitro.^224^ However, from an in-cell NMR analysis no metal binding was observed in human cells treated with either zinc or copper. Conversely, Cys106 was oxidized to sulfinic acid upon treatment of cells with H_2_O_2_, thus confirming its redox sensor activity.^91^

As mentioned previously, an SOD1 disulfide bond contributes to structurally stabilize the protein, and its formation prevents SOD1 from misfolding and aggregating. For this reason, the cysteines involved in this bond could represent a potential target for novel therapeutic treatments of ALS patients. By in-cell NMR experiments it was observed that the organoselenium compound ebselen is able to interact with Cys57 and Cys146 allowing the complete oxidation of E,Zn-SOD1 through a seleno-thiol exchange mechanism. The experiments were also repeated in cell-expressing ALS-related SOD1 mutants, and, interestingly, samples treated with ebselen showed E,Zn-SOD1 oxidized levels comparable to those of the WT SOD1, while the untreated samples presented a large accumulation of unfolded, reduced species. This indicates that ebselen, acting as a CCS analogue, contributes to helping SOD1 reach a correct folding and to prevent possible cytosolic aggregation of the immature or mutant species of the protein.^225^

An external stimuli that is related to the alteration of cellular redox homeostasis is the exposure to cadmium.^226^ This impairing of the redox pool is probably due to the replacement of many metal ions from their native binding sites thus damaging mitochondria and making ineffective most of the antioxidant proteins.^227^ In particular, cadmium negatively affects the enzymatic activity of SOD1.^228^ The effect of cadmium treatment on the metal and redox state of SOD1 was investigated by NMR in HEK293T cells by Luchinat and Banci.^229^ With a cadmium treatment, the induction of a massive expression of the metallothionein (MT) isoforms MT-1X and MT-2A was observed, up to NMR-detectable levels. Concurrently, SOD1 did not show any evidence of cadmium binding to either the zinc or copper binding sites. Interestingly, it was noted that, in cells treated with an excess of Zn, E,Zn-SOD1 was mostly in the reduced form and the two MT isoforms were highly expressed; instead, in a defect of Zn, the oxidized form of E,Zn-SOD1 prevailed, and there was a lower, albeit significant, expression of MT-1X and MT-2A. The presence of cadmium in the cellular environment causes the displacement of zinc ions from MTs and other proteins, thus increasing the amount of available zinc in the cell. In turn, these “liberated” zinc ions bind to and activate the metal-responsive transcription factor (MTF-1), inducing the overexpression of MTs, which bind cadmium and protect the cells from further damage. It was hypothesized that, at basal levels of zinc, overexpressed SOD1 seizes all the zinc ions displaced by cadmium, thus impeding the activation of MTF-1. The lower induction of MTs results in an impaired redox balance with the consequent premature SOD1 disulfide bond formation. Instead, with an excess of zinc, SOD1 metalation does not prevent MTF-1 activation, and the consequently higher expression of MTs prevents the redox imbalance and the oxidation of SOD1.

#### 3.4.3 Post-Translational Modifications

Phosphorylation is a common post-translational modification occurring in the cellular environment, playing a fundamental role in many cellular processes like signaling events, activation of enzymes, and cellular proliferation and is proven to be fundamental for the function of many proteins.^230−232^ In one of the landmark advancements of the in-cell NMR methodology reported by Selenko and Wagner, the entire phosphorylation pattern performed by casein kinase 2 (CK2) on its substrate, the viral SV40 large T antigen regulatory region, was analyzed by time-resolved NMR experiments conducted in whole living *X. laevis* oocytes, in oocyte extracts and in vitro. The resulting spectra revealed that the series of phosphorylation processes occurred in a defined order, with the release of intermediate substrates.^233^ The Lippens group analyzed the phosphorylation pattern of Tau in *X. laevis* oocytes, which turned out more challenging due to its size and the large number of possible phosphorylation sites.^234^ A similar approach was adopted to study the multiple phosphorylation/dephosphorylation events on the unique domain of C-Src, a member of the nonreceptor tyrosine kinases.^235^ Through real-time NMR, Amata et al. were able to characterize the phosphorylation of Ser17 directly in living oocytes and to follow the phosphorylation of other C-Src residues in egg extracts. Furthermore, through the analysis of the effects of different kinase inhibitors, they identified the specific enzymes responsible for the phosphorylation of each specific residue and highlighted the mutual interplay between kinases and phosphatases. These studies proved that a time-resolved in-cell NMR approach is the technique of choice for easily detecting and monitoring multiple phosphorylation events directly in living cells. Indeed, protein resonance signals are strongly perturbed by phosphorylation, thus making NMR a suitable tool for an investigation of this kind of post-translational protein modifications. More recently, Theillet et al., as a part of a seminal study focused on the investigation of the intracellular conformation and dynamics of α-syn, showed that the physiological state of α-syn in human cells is acetylated at the N-terminus. Notably, the N-terminal acetylation reaction occurred in a post-translational fashion, as delivering nonacetylated α-syn to the human cells resulted in the complete formation of a N-acetylated protein.^39^

### 3.5 Protein Dynamics

The dynamical properties of biomolecules play a key role in determining the function of biomolecules. Indeed, molecular tumbling, conformational rearrangements, and domain reorientations are essential for partner interactions and, together with internal motions, to exert their physiological activities. Biomolecular motions occur over a broad time range. The overall molecular tumbling is determined by the molecular size, in addition to viscosity and obviously to temperature. Vibrational motions occur in the range of picoseconds/nanoseconds, while conformational changes usually occur with time scales from microseconds up to seconds.^236^ NMR has proved to be one of the most powerful approaches for characterizing biomolecular dynamics, as the spin relaxation properties are determined by the motions they are involved in. Longitudinal and transverse relaxation rates (*R*_1_ and *R*_2_) and the heteronuclear NOE (hetNOE)^237^ are the most common experiments used to estimate the extent and time scales of the internal motions as well as of the overall molecular reorientation.

The analysis of protein motions inside living cells can be fundamental for understanding how the physiological environment affects such molecular dynamics, although recording spin relaxation experiments on living cells is challenging. Indeed, high-sensitivity spectra are required and, in a cell, the environment viscosity, nonspecific interactions, and the presence of background signals are limiting factors for these measurements. Despite these limitations, in-cell NMR has provided relevant insights into the dynamics of intracellular proteins, especially when applied to the study of the conformational dynamics of IDPs. The Pielak group provided clear empirical proof that IDPs suffer less from the drawbacks mentioned above when compared to globular proteins, by analyzing α-syn and a globular protein, the chymotrypsin inhibitor 2 (CI2), in bacteria. CI2 proved to be invisible to NMR due the effect of the crowded cellular cytoplasm, whereas α-syn was clearly detected.^238^ The same authors then observed in bacteria an artificial construct in which ubiquitin was fused to α-syn: only the NMR signals from α-syn were detected, thanks to its internal motions being independent from the slow tumbling of ubiquitin.^239^

**Figure 4:**
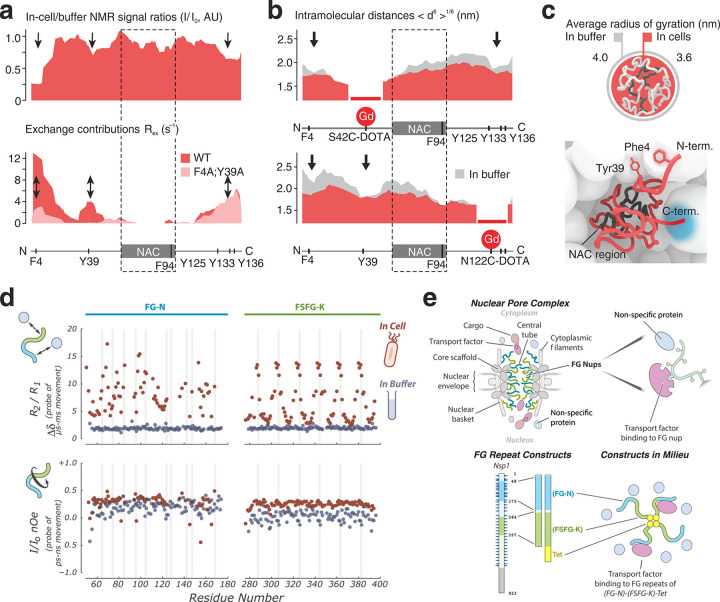
Conformational dynamics of IDPs probed by in-cell NMR. (a–c) The dynamics of α-syn in human-derived A2780 cells: (a) in-cell/in vitro relative signal intensity (*I*/*I*_0_, top) and exchange contribution (*R*_ex_, bottom) plotted for each residue of α-syn; (b) intramolecular PRE-derived distance profiles α-syn in buffer (gray) and in A2780 cells (red). Regions with decreased intensity, increased exchange, or increased compaction are indicated with arrows. (c) Intracellular α-syn is more compact (top) and interacts with cellular components at the N-terminus through hydrophobic residues and electrostatically at the C-terminus (bottom). Reproduced with permission from Theillet et al.^39^ Copyright 2016 Nature Publishing Group. (d, e) The dynamics and interactions of FG Nups in bacteria: (d) in-cell NMR relaxation and hetNOE measurements on two FG Nups regions, plotted for each residue, compared to in vitro data. The locations of the FG motifs are indicated with gray bars. (e) The location of FG Nups within the nuclear pore complex, and the main features of the FG-Repeat constructs analyzed by in-cell NMR. Reproduced with permission from Hough et al.^142^ Copyright 2015 Hough et al.

In-cell NMR was extensively employed to characterize the conformational dynamics of α-syn, both in *E. coli* and in human cells. Croke et al. collected NMR relaxation and chemical exchange experiments to study the α-syn conformational states both in vitro and in *E. coli*.^146^ In contrast to what was previously suggested, the authors demonstrated that the loss of α-syn signals observed in vitro at physiological temperatures and pH is due to a fast proton amide exchange with the solvent, rather than to a conformational exchange. In fact, by the evaluation of Cα chemical shifts it was found that the α-helix structured α-syn did not reach the 10% of the total protein amount at temperature conditions between 10° and 35 °C, showing that temperature does not affect its conformational state. In bacteria, the amide exchange was found to be slower, likely because of a more acidic environment than expected, thus explaining the permanence of in-cell NMR signals observed in previous studies and confirming that intracellular α-syn is fully disordered. Similar results were obtained by Binolfi et al., who analyzed the C′ chemical shifts in *E. coli* and found that they matched those of α-syn in vitro, which is in a completely disordered state.^58^ Waudby and coauthors employed signal deconvolution to improve the accuracy of backbone chemical shift measurements and evaluated the distribution of secondary structure populations of α-syn in bacteria. Consistent with the above observations, α-syn was found to exist in a highly dynamical, disordered conformation and showed only minor shift changes with respect to that in vitro, which were attributed to the interaction with cellular components.^165^ In an extensive study that combined in vitro and in-cell NMR and EPR spectroscopies, the groups of Selenko and Goldfarb provided the first insights into the conformation and dynamics of α-syn in non-neuronal and neuronal mammalian cell lines.^39^ In all the cellular types, α-syn was found to be N-terminal acetylated. Like in bacteria, also in human cells α-syn remained fully disordered and monomeric. The dynamic properties of intracellular α-syn were then investigated by NMR relaxation, showing that weak and transient hydrophobic and electrostatic interactions with intracellular partners affect the dynamics of various regions of the protein differently (Figure 4a). Furthermore, NMR paramagnetic relaxation enhancement (PRE) and EPR measurements showed that α-syn takes a more compact conformation in cells than in vitro, with potentially relevant implications on the pathogenic aggregation pathway (Figure 4b,c). The above results were instrumental to settle a debate on the existence of a helical, tetrameric state of α-syn in solution and in cells^240,241^ and to demonstrate that in-cell NMR is the method of choice to probe the conformational dynamics of IDPs in the relevant cellular settings.

Another highly dynamic system that was investigated by in-cell NMR is the transport across the nuclear membrane, which is regulated by a selective filter within the nuclear pore complex (NPC).^142,242,243^ The import/export of macromolecules through the NPC is regulated by their interaction with specific proteins known as transport factors (TFs). TFs can cross the NPC by the reversible association with nucleoporins lining the inner side of the NPC. Some nucleoporins consist of long intrinsically disordered regions that are rich in phenylalanine-glycine (FG) repeats, so-called FG Nups (Figure 4e).^244,245^ Hough et al. investigated the dynamic behavior of FG Nups by NMR both in vitro under crowded conditions and in the cellular environment and showed that the interactions with the intracellular milieu are essential for maintaining FG Nups in a disordered and highly dynamics state. Indeed, an FG Nups aggregation was observed under noncrowded conditions, whereas high-molecular weight complexes were not detected in *E. coli*, thus suggesting that cell cytoplasm worked as an inhibitor of the intermolecular FG repeat aggregation.^142^ Notably, the hydrophobic residues responsible for the contacts with transport factors were found to be mainly involved in the interactions with intracellular partners (Figure 4d). In a following study, the same group analyzed a fragment of the Nsp1 FG Nup, known as FSFG-K, in the yeast *S. cerevisiae*.^66^ The fragment is disordered and highly dynamic in the yeast cytoplasm, consistent with what was observed in bacteria. However, some differences were observed at the residue level: the second phenylalanine in the FSFG motifs showed a markedly increased transverse relaxation rate, suggesting that, while transient interactions are present in both environments, the interactions in yeast have different specificities within the FG repeat. The above results suggested a mechanism for the selective diffusion through the NPC, where the highly dynamic state of nucleoporins is fundamental to increase the diffusion of TFs,^242,243^ in contrast to the hypothesis that nucleoporins exist in a gel-like state that dissolves locally to allow the passage of TFs.^246,247^

Recently, the Blackledge group reported an approach that, by collecting hetNOE and longitudinal, transverse, and cross-correlated dipole–dipole/CSA ^15^N relaxation data in a broad range of crowding conditions in vitro, provided a unified description of the dynamic behavior of IDPs in complex environments, such as the cytoplasm, with only a small set of known physical parameters. The authors validated the approach by investigating the dynamics of the disordered N-terminal domain of the mitogen-activated kinase 4 in *X. laevis* oocytes, reporting a good agreement between the predicted and experimentally derived dynamic behavior.^248^

Similar to what was observed for IDPs, the cellular environment can also affect the local conformational dynamics of folded proteins. However, examples of such an application to folded proteins are scarce, as the main focus was to understand how the cellular milieu affects the overall tumbling of folded macromolecules (see section 3.1). Shirakawa and Hamachi provided an example of how in-cell NMR can probe local protein dynamics, by comparing the ligand-bound and unbound forms of human carbonic anhydrase I (CA I) dynamics in intact erythrocytes and in vitro.^156^ The authors employed a ligand-directed tosyl (LDT) approach^249^ to tether a fluorinated probe to CA I directly inside the erythrocytes without affecting the protein native folding. The rate of exchange between free CA I and CA I bound to benzenesulfonamide, obtained by ^19^F EXSY experiments, was 1.6-fold faster in red blood cells than in vitro. Furthermore, the intracellular protein showed larger conformational fluctuations suggesting that this dynamics enhancement may have a major role in facilitating the substrate/product release.

### 3.6 Ligand Screening

The capability of NMR to extract information about molecular interactions at the atomic level has made this technique extremely powerful in the field of drug discovery. Two complementary approaches are exploited to screen protein–ligand interactions by NMR: ligand-observed and protein-observed screening. Through ligand-observed NMR, large libraries of compounds are typically screened with respect to their ability to bind a given target. Instead, a protein-observed analysis provides detailed information on the mode of binding of molecules to the target and on structural properties of the adduct. Approaches based on ligand detection include saturation transfer difference (STD)^250^ and WaterLOGSY,^251^ which are based on a magnetization transfer to detect a ligand binding, or simple ^1^H NMR relaxation measurements,^252^ which detect changes in the average molecular mobility of the ligands upon interaction with the target.^253^ In protein-observed approaches, a chemical shift perturbation analysis is performed on heteronuclear 2D NMR spectra recorded on the protein at increasing ligand concentrations. In addition to assess whether the molecule binds the target or not, the CSP data provide, with very high accuracy, information on which functional groups or residues are involved in the binding. The approach based on the structure–activity relationship by NMR (SAR by NMR)^254^ has been intensively used for monitoring ligands with low affinity and for collecting the structural information needed to enhance their specificity.^255^

All the above-mentioned techniques have been developed and applied essentially as in vitro assays, and therefore they present some limitations. In fact, the absence of the cellular context limits the study to just the candidate drug and the target. While an in vitro screening remains useful to discard low-affinity or non-interacting compounds, it does not take into account the potential interactions with other cellular components as well as whether the drug is able to pass through the plasma membrane. In-cell NMR represents the ideal method for probing at the structural level protein–ligand interactions within living cells. Therefore, potentially, it could contribute to overcoming common bottlenecks encountered at more advanced stages of the drug discovery pipeline, such as low tissue availability or poor target selectivity, which often translate to drug efficacy or safety issues that determine a large part of clinical failures.^256^ Several ligand-observed and protein-observed in-cell NMR drug screening approaches have been developed, to improve both the technique throughput and the range of possible applications.

#### 3.6.1 Protein-Observed Screening

**Figure 5:**
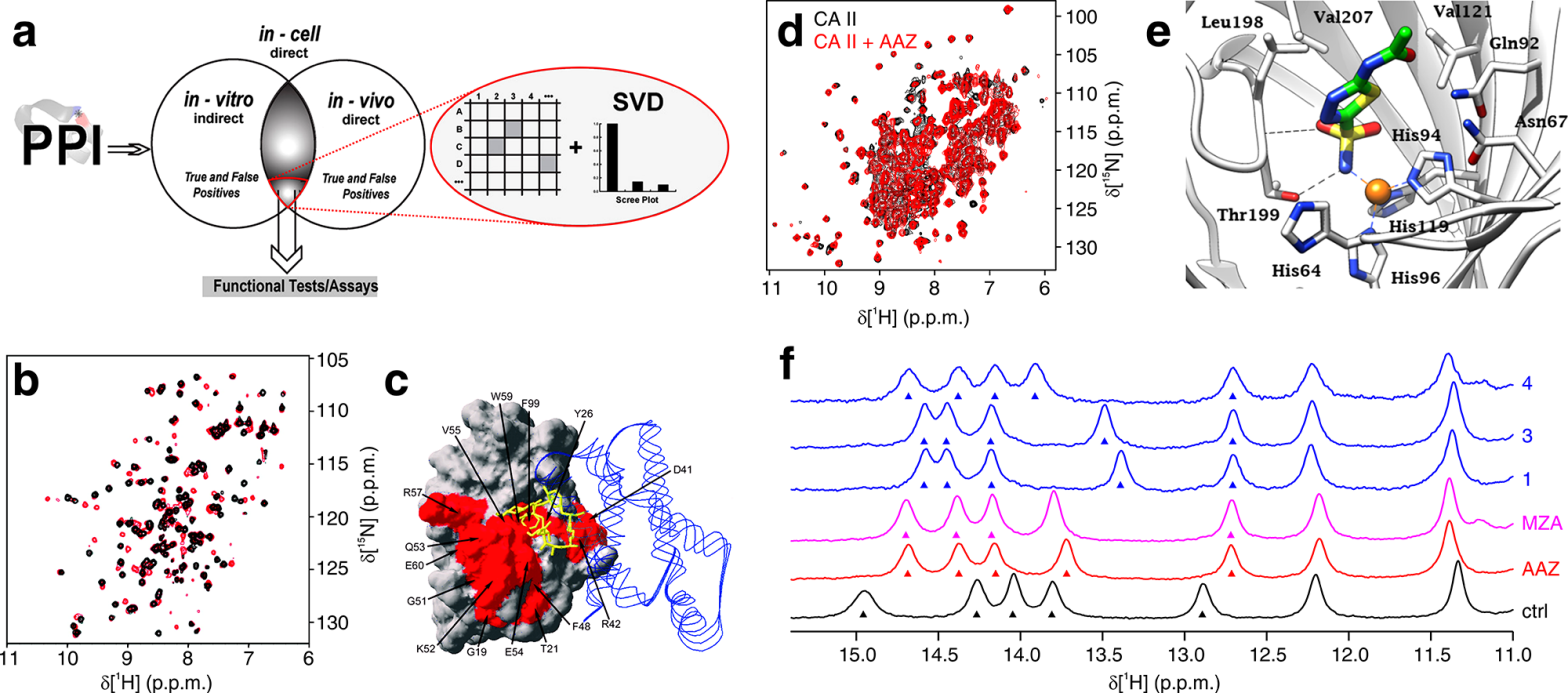
Protein-observed intracellular ligand screening approaches. (a) HTS for compounds that disrupt protein–protein interactions (PPIs) using in-cell NMR combines the advantages of in vitro and in vivo studies by providing residue-specific information in a physiologically relevant environment. Spectra from samples treated with mixtures through the matrix approach are analyzed by SVD. Adapted with permission from DeMott et al.^168^ Copyright 2018 American Chemical Society. (b) ^1^H–^15^N in-cell NMR spectra of FKBP in bacteria in complex with unlabeled FRB in the absence (black) and presence (red) of rapamycin. (c) Surface residues of FKBP involved in the interaction with rapamycin; FRB is shown in blue. Reproduced with permission from Xie et al.^257^ Copyright 2009 American Chemical Society. (d) ^1^H–^15^N in-cell NMR spectra of CA II in human cells in the absence (black) and presence (red) of acetazolamide (AAZ); (e) 3D view of AAZ bound to the catalytic zinc ion in the active site of CA II (PDB: 3HS4). (f) Imino region of the 1D ^1^H NMR spectra of CA II in human cells in the absence of ligands (black) and treated with AAZ (red), MZA (magenta), and other ligands (blue). Reproduced with permission from Luchinat et al.^92^ Copyright 2020 Luchinat et al.

After the early in-cell NMR works demonstrated the application to protein–ligand interactions, the first in-cell NMR-based high-throughput screening (HTS) procedure was developed by the Shekhtman group (Figure 5a).^257^ The approach, termed SMILI-NMR, was based on the STINT-NMR technology previously developed by the same group (see section 3.2)^192^ and, in principle, allows efficient screening of entire libraries of compounds by identifying those able to induce structural changes by binding to a protein–protein complex in bacteria. The heterodimer constituted by FKBP and FRB, important for the modulation of immune response in humans,^258^ was used as the test system. The two proteins were sequentially overexpressed in *E*. *coli* following the STINT-NMR protocol and analyzed by ^1^H–^15^N HSQC. ^15^N-FKBP alone did not give rise to detectable signals in cells, whereas after the expression of unlabeled FRB and subsequent complex formation, FKBP gave rise to well-resolved peaks (Figure 5b). Incubation with rapamycin or ascomycin, antibiotics that enhance the FKBP-FRB complex formation by interacting with FKBP, resulted in the formation of a ternary complex, and a CSP analysis allowed one to identify the interaction surface (Figure 5c). A library of 289 dipeptides arranged in a 17 × 17 matrix was then screened by monitoring their effect on the in-cell NMR spectrum of the complex. Drugs placed in one row or in one column of the matrix plate were mixed and added to one cell sample, greatly reducing the number of samples required. Single compounds at the intersection of row and column hits were subsequently screened to prove their efficiency.^257^ The same group further applied SMILI-NMR to screen a library of ∼1600 compounds to identify potential antimicrobial agents against *Mycobacterium tuberculosis*. The compounds were screened for their ability to inhibit the interaction between the prokaryotic ubiquitin like protein (Pup) and mycobacterial proteasome ATPase (Mpa) expressed in bacteria.^168^ The Pup-Mpa complex is deemed fundamental for the resistance of the bacterium against nitric oxide stress (see section 3.2), thus representing a potential target for novel drugs. Three compounds were identified that efficiently inhibit the complex formation, which could then inhibit the growth of *Mycobacterium* under nitric oxide stress with an efficacy comparable to that of rifampicin.

The first application of in-cell NMR to drug screening in human cells was reported by the Banci group. The interaction between the second isoform of carbonic anhydrase (CA II) and a series of CA inhibitors was monitored through protein-observed NMR in human cells.^92^ The spectral changes induced by the binding of two reference compounds, the approved drugs acetazolamide and methazolamide, were monitored by ^1^H–^15^N 2D NMR (Figure 5d,e). Furthermore, protein signals in the background-free imino region of the ^1^H 1D spectra allowed the screening of newly developed compounds without resorting to isotopic labeling (Figure 5f). A quantitative analysis of the intracellular binding was performed on a subset of cell-penetrant molecules, for which the fitting of dose- and time-dependent binding curves provided relevant parameters related to the membrane permeability and intracellular binding affinity.^92,172^ The application of such an approach in the frame of the traditional drug design pipeline could provide important insights on the intracellular binding specificity of approved drugs, as shown by the same authors on a set of molecules originally developed for different targets and later found to inhibit CA through off-target binding.^172^ That study revealed strikingly different behaviors, as some drugs showed binding instability over time, possibly as a consequence of the high affinity toward other intracellular targets.

#### 3.6.2 Ligand-Observed Screening

Ligand-observed screening approaches have also been developed. From a methodological point of view, while the above protein-observed screening approaches are a direct application of macromolecular in-cell NMR, observing ligands in living cells by NMR often eludes the “in-cell NMR” label, having substantially different requirements in terms of cell types and density, expression levels of the intracellular target, and type of NMR experiments. Several studies have been reported where compounds were screened for binding to target receptors on the surface of intact cells.^259−261^ However, these studies focus on surface receptors and are based on the assumption that the ligand is selective for the target protein; that is, the target engagement is not specifically assessed.

Bouvier et al. applied in-cell NMR to validate target engagement of compounds belonging to the antituberculosis imidazopyridine amide (IPA) family.^262^ The nonpathogenic strain, *Mycobacterium smegmatis*, overexpressing the putative IPA drugs target, that is, *M. tuberculosis* cytochrome complex (QcrCAB_Mbt_), was used for the validation. Ligand-based ^1^H STD experiments showed the occurrence of drug binding to the Cytochrome *b* subunit, providing a detailed model of the interaction surface between an IPA drug and its target. Primikyri et al. developed a similar approach for mapping the binding of ligands to an intracellular target in human cells.^263^^1^H STD and transferred-NOE spectroscopy (Tr-NOESY) experiments were applied to map the binding of a quercetin-alanine bioconjugate to the antiapoptotic protein Bcl-2 inside living human T-leukemic cells stably expressing the target protein. The target engagement was further validated with in vitro protein-observed NMR data on ^15^N-labeled Bcl-2. Although promising, currently these ligand-observed in-cell NMR approaches have yet to find a wider application to ligand screening in living cells.

In addition to the approaches outlined above, where the ligands binding to the targets are directly investigated (so-called on-target methodologies), off-target methodologies have also been developed, which indirectly probe protein–ligand interactions by evaluating the effect of ligand binding on the substrate of an enzymatic reaction. Notably, here the observed molecule typically does not interact directly with the drug target, and therefore NMR is not employed to detect an intermolecular interaction but to monitor the rate of enzymatic reaction in real time.

The off-target approach was used in drug screening studies coupled with the employment of ^19^F NMR. The observation of fluorinated compounds has proved its versatility and applicability in many drug discovery research projects.^264,265^ In particular, the Dalvit group developed a ^19^F in-cell NMR methodology termed n-fluorine atoms for biochemical screening (nFABS) to identify inhibitors for relevant pharmaceutical targets in living cells.^266^ In that work, the inhibition of a specific enzymatic target, the membrane protein fatty acid amide hydrolase (FAAH), was assessed by observing the cleavage of a fluorinated substrate, the anandamide analogue ARN1203, in human HEK293 cells. In addition, the combination of ^1^H and ^19^F NMR allowed one to build a metabolic fingerprint of the cells, thus making it possible to evaluate possible metabolic changes caused by the tested compounds. A similar approach relying on the observation of the substrate of an enzymatic reaction by ^1^H NMR was reported by Ma et al.^267^ With that approach, potential inhibitors of the New Delhi metallo-*b*-lactamase subclass 1 (NDM-1) enzyme, involved in the bacterial defense mechanism against antibiotics, were screened in bacteria. The activity of NDM-1 was monitored through time-resolved ^1^H NMR by observing the decrease of signals from the substrate, Meropenem, and the simultaneous increase of signals from the product, in the presence of different inhibitors.

### 3.7 Nucleic Acid Conformation, Stability, and Interactions

**Figure 6:**
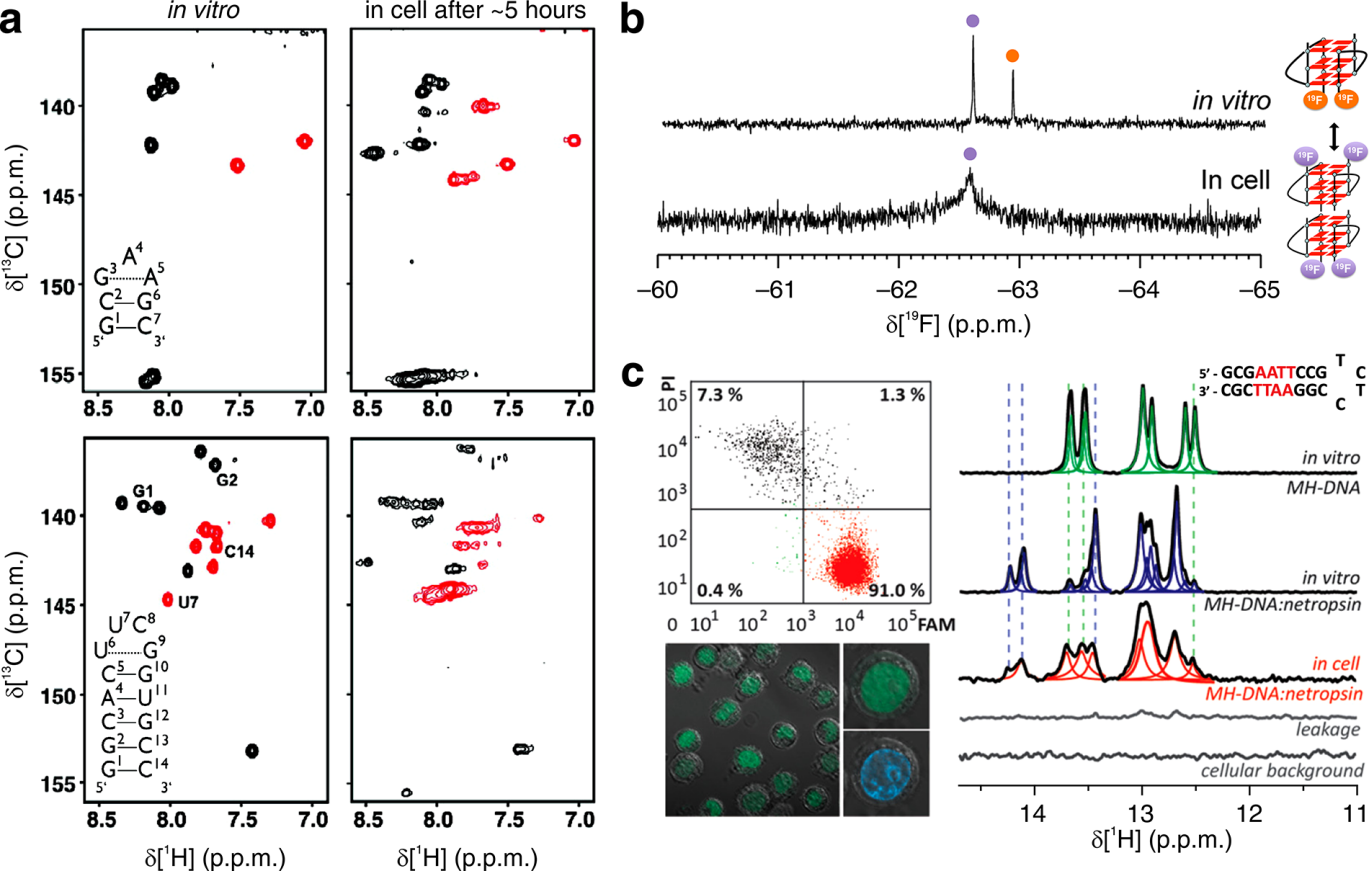
Structure and interactions of nucleic acids revealed by in-cell NMR. (a) ^1^H–^13^C NMR spectra of isotopically labeled DNA (top) and RNA (bottom) hairpins recorded in vitro (left) and in *X. laevis* oocytes 5 h after a microinjection. The hairpin structures are shown in the left panels. Adapted with permission from Hänsel et al.^102^ Copyright 2009 American Chemical Society. (b) Different topologies of telomeric RNA G-quadruplex observed by ^19^F NMR experiments in vitro (top) and in *X. laevis* oocytes (bottom). The ^19^F signals corresponding to the G-quadruplex dimer (orange) and two-subunits stacked G-quadruplex (violet) are color-coded according to the structures (right). Adapted with permission from Bao et al.^101^ Copyright 2017 Bao et al. (c) Interaction of a DNA hairpin (MH-DNA) with netropsin in human cells: (top left) flow cytometry analysis after electroporation; viable MH-DNA containing cells are shown in red. (bottom left) Localization MH-DNA (green) inside the cell nucleus (blue). (right) Deconvoluted imino region of MH-DNA 1D ^1^H NMR spectra obtained alone and in the presence of netropsin in vitro (green and blue) and in human cells (red). Reproduced with permission from Krafcikova et al.^106^ Copyright 2019 American Chemical Society.

The use of in-cell NMR to study nucleic acids has opened the possibility to investigate their structure and their interactions inside the eukaryotic cellular environment. While the first applications reported were limited to *X. laevis* oocytes, delivery approaches were later developed for mammalian cell cultures (see section 2.1.3). In-cell NMR was first applied to nucleic acids by Trantirek, in collaboration with Dötsch and Schwalbe.^102^ That work, which set the basis for subsequent in-cell NMR investigations, investigated the conformation adopted by a G-quadruplex in intact oocytes. The G-quadruplex is arguably one of the most important and studied DNA structural motifs,^268^ since the discovery of G-quadruplex repeats in the telomeric DNA inhibiting the activity of telomerase,^269^ an enzyme involved in the proliferation of tumoral cells.^270^ In vitro, G-quadruplexes are very sensitive to the environmental conditions and, specifically, to the concentration of monovalent cations.^271^ Hänsel and coauthors injected an unlabeled d(G_3_(TTAG_3_)_3_T) DNA motif in oocytes and observed that the signals in the 1D ^1^H NMR spectra differed from the typical pattern of basket-type-G-quadruplex observed in vitro in the presence of K^+^ ions,^272^ due to the occurrence of extensive line broadening. Narrower NMR signals were observed in oocyte extracts obtained by denaturing the protein fraction, where a mixture of DNA conformations was observed, while an extraction of the intraoocyte DNA molecule and a subsequent NMR analysis excluded intracellular degradation. It was therefore hypothesized that low-molecular-weight cellular components played a crucial role in promoting the folding conformation polymorphism assumed by telomeric G-quadruplexes. In the same work, it was also shown that labeled DNA and RNA hairpin motifs, either unmodified or stabilized with phosphorothioate esters, could be observed in oocytes and exhibited a sufficiently long half-life for NMR spectra acquisitions (Figure 6a).^102^

In-cell NMR in oocytes was applied to investigate interactions of nucleic acids with ligands. Salgado et al. studied the binding of a ligand, 360A, a golden standard for its proved affinity and specificity for telomeric DNA G-quadruplex,^273^ to a G-quadruplex motif, d(TG_4_T)_4_, in oocytes.^111^ Ligand binding was observed both upon incubating preinjected oocytes with the ligand or by injecting the preformed adduct. The intracellular adduct gave rise to a different pattern of signals in oocytes with respect to that observed in vitro, whereas the spectrum of the lysate was consistent with the one recorded in vitro, confirming the complex formation. Although the data were insufficient to provide more information on the in-cell conformation, the work showed that in-cell NMR could monitor the interaction between nucleic acids and ligands.^111^

The telomeric RNA G-quadruplex topology was investigated more recently in vitro and in intact oocytes by Bao et al.^101^ The authors employed in-cell ^19^F NMR to address whether telomeric repeat-containing RNA molecules could form a high-order structure in cells, in which two G-quadruplex subunits are stacked together.^274,275^ An r(UAGGGUUAGGGU) RNA fragment, ORN-1, was tagged with fluoromethylbenzene at the 5′-end and injected into oocytes. The ^19^F chemical shift of the intracellular RNA was comparable to that observed in vitro and consistent with a high-order RNA G-quadruplex structure (Figure 6b). The same dimeric G-quadruplex was observed in vitro at 0.5 mM RNA concentration, suggesting that the structure observed in oocytes was not an artifact caused by the highly concentrated RNA solution injected (3–5 mM). An in vitro analysis in a crowded solution indicated that the formation of a high-order RNA G-quadruplex was promoted by molecular crowding.^101^

Similar to proteins, nucleic acid motifs involved in human diseases should be studied in a more relevant context, that is, in human cells. In fact, telomeric DNA G-quadruplex ligands are pharmacologically relevant in anticancer treatment,^276^ and, therefore, methods to investigate them in a human cell environment can provide precious insights for future therapeutic developments. The first observation of in-cell NMR signals of nucleic acids in living human cells was independently reported by the Katahira and Trantirek groups (see section 2.1.3). Yamaoki et al. delivered an oligo-DNA (5′-G*C*GAAGC-3′, *: phosphorothioate) and an oligo-RNA (5′-GGCACUUCGGUGCC-3′, fully 2′-OMe) into HeLa cells using the pore-forming toxin SLO approach.^104^ Both molecules were shown to form stable hairpins in cells, similar to what was observed in vitro. Dzatko et al. investigated a series of oligo-DNA sequences known to form in vitro four-stranded structures called i-motifs, the biological relevance of which was disputed at the time, due to the lack of evidence for their existence in vivo.^277^ The oligo-DNAs were delivered in HeLa cells by electroporation and were localized in the nucleus, where the presence of i-motifs and their structural stability were investigated. Not only were i-motifs present in the human cell nucleus but they even showed an increased stability at physiological temperatures than in vitro, likely due to the interaction with cellular components.^105^

The Trantirek group moved further and applied the above approach to the investigation of DNA-ligand interactions in human cells. Krafcikova et al. electroporated in HeLa cells preformed complexes between a 24-nt DNA hairpin (MH-DNA) and netropsin, a minor groove-binding compound, and between a 11-bp DNA duplex with a T·T mismatch (TT-DNA) and three naphthalenophane-based compounds specific for DNA base-pairing defects (Figure 6c).^106^ 1D ^1^H in-cell NMR revealed that, while the MH-DNA:netropsin adduct and the TT-DNA in complex with the first two drugs were stably formed in cells, the third ligand dissociated from TT-DNA. An NMR analysis of the same complex in a cytosolic extract, intact cell nuclei, small metabolite cellular fraction, and in buffer in the presence of mimics of genomic DNA off-targets, suggested that some metabolites competed against the third compound for TT-DNA binding, leading to dramatic changes in the signal patterns of the complex.

The Petzold group showed that the behavior of oligonucleotide-based candidate drugs can be studied in human cells by NMR.^278^ Schlagnitweit et al. focused on a 16-nucleotide synthetic antisense oligonucleotide (ASO) with a phosphorothioate backbone, known to downregulate the STAT3 transcription factor mRNA and thus exert antitumoral effects in different types of cancer.^279,280^ ASO was delivered into HeLa and HEK293T cells either through electroporation or through free uptake. In both cases, real-time quantitative polymerase chain reaction (qRT-PCR) showed downregulation of STAT3 mRNA, confirming the correct cellular uptake. However, no signals from intracellular ASO were detected in the ^31^P in-cell solution NMR spectra, likely due to the formation of large complexes. Consistently, ASO was detected in the NMR spectra of cell lysates upon enzymatic digestion of the protein fraction. To overcome the molecular tumbling limit and the low sensitivity of solution NMR, the authors relied on dynamic nuclear polarization (DNP)-assisted solid-state NMR on cryoprotected frozen cells, where they successfully detected ∼15 μM intracellular ASO. Cell freezing conditions were optimized to allow the cells to remain viable when reseeded after the NMR experiments. The application of DNP can thus enable the NMR investigation of nucleic acids involved in large complexes within the cells.

Very recently, in the continuous effort to extend the applicability of in-cell solution NMR to a greater variety of systems, Broft et al. successfully characterized larger and more complex RNA molecules.^103^ In particular, they were able to evaluate the stability, the structure, and the interaction with ligands of different aptamer domains and of an RNA hairpin both in oocytes and HeLa cells. They demonstrated the possibility of using in-cell NMR for studying a nonmodified RNA strand and for detecting the 2′-deoxyguanosine binding to a prokaryotic riboswitch in eukaryotic cells. At the same time, it increased the previous ∼15 nucleotides size limit of RNA fragments amenable to an in-cell NMR analysis, by characterizing an ∼70 nucleotides-long molecule.

Overall, the above studies show how in-cell NMR can provide structural insights on intracellular nucleotide structural motifs, on DNA-ligand complexes, and on oligonucleotide-based drugs. It can also evaluate their stability and assess the binding of other cellular components, thus providing a new useful methodology in the DNA-drug discovery pipeline.

## 4 Bioreactors for In-Cell NMR

As seen in the previous sections, in-cell NMR spectroscopy has proved to be a powerful technique to investigate conformational and functional properties of macromolecules within a physiological cellular environment. Despite the continuous development of new approaches to extend the in-cell NMR applicability to more complex types of cells, the intrinsic poor sensitivity of NMR spectroscopy and the short lifetime of the cell samples remain big limiting factors. These two problems are interlinked: to overcome the first, that is, to increase the NMR sensitivity and record experiments in a shorter time, the cells need to reach very high densities in the NMR tube. In turn, high cell densities result in a faster depletion of oxygen and nutrients and an accumulation of waste metabolites, causing the progressive decrease of cell culture viability. To overcome this problem, bioreactor devices, which can greatly increase the cellular lifetime and are able to fit the NMR spectrometer, have been developed during the years. These devices not only allow one to monitor biological processes with a higher sensitivity but make it possible to observe them as they occur in real time.

In fact, devices to keep cells alive in NMR spectrometers have been developed since the 1980s, either in the form of fermenters that allowed a continuous observation of bacterial and yeast cell cultures or in the form of perfusion systems for the analysis of mammalian cells. Some of these bioreactor designs were highly complex and enabled precise control of pH, media composition, and concentration of dissolved gases. However, they were designed for wide-bore magnets equipped with NMR probes that allowed fitting tubes, typically 20 or 10 mm, wider than the 5 or 3 mm probes more common today. These wider bioreactors could grow large biomasses and were mainly applied to study metabolic fluxes as a function of various cell-growth parameters. With the advent of macromolecular in-cell NMR spectroscopy, the concept of NMR bioreactor was rediscovered, but new devices had to be developed that could fit modern, narrow-bore ultrahigh field magnets while still maintaining high cell densities in order to maximize the NMR sensitivity.

### 4.1 Bioreactor Systems

The wide-bore NMR bioreactors developed for a metabolic analysis of cell cultures made use of different principles to bring the cells into the NMR spectrometer while ensuring a proper exchange of growth media. Different bioreactor designs were introduced that were optimized for either suspended or adherent cells. For cell cultures that grow in suspension, including bacteria and yeast, bioreactors that allow a continuous circulation of the suspension cell culture between a reactor vessel and the NMR magnet represent a valid method. Such devices were developed by different research groups and were based on a similar scheme. In the system developed by de Graaf et al., bacterial cells grown in suspension in a reactor vessel were fluxed by a pump into a measuring chamber constituted by the final portion of a 20 mm NMR tube.^281^ On the basis of the same principle, Meehan et al. employed an air turbine to allow the circulation of a yeast culture.^282^ These devices could be equipped with an oxygenation apparatus, pH probes, and valves to add reagents. Chen and Bailey built an online NMR spectroscopy system in which a pump flowed a bacterial suspension cultured in a fermenter to a 20 mm NMR tube.^283^ The online bioreactor included an aeration system that allowed to switch the cultivation conditions between aerobic and anaerobic by simply replacing oxygen with nitrogen.

A different kind of system, in-magnet bioreactors, was designed for a continuous growth of microorganisms confined within the NMR magnet, keeping them metabolically active over a prolonged period of time, allowing for NMR experiments at high microbial cell densities.^284,285^ Among these, the 20 mm wide membrane cyclone reactor developed by Hartbrich et al. was made to operate continuously inside the magnet and proved to achieve higher cell densities compared to the continuous circulation systems described above. Later, Majors et al. used a similar NMR bioreactor design to maintain anaerobic bacteria in controlled growth conditions for the analysis in an imaging spectrometer fitting 20 mm tubes.^285^

**Figure 7:**
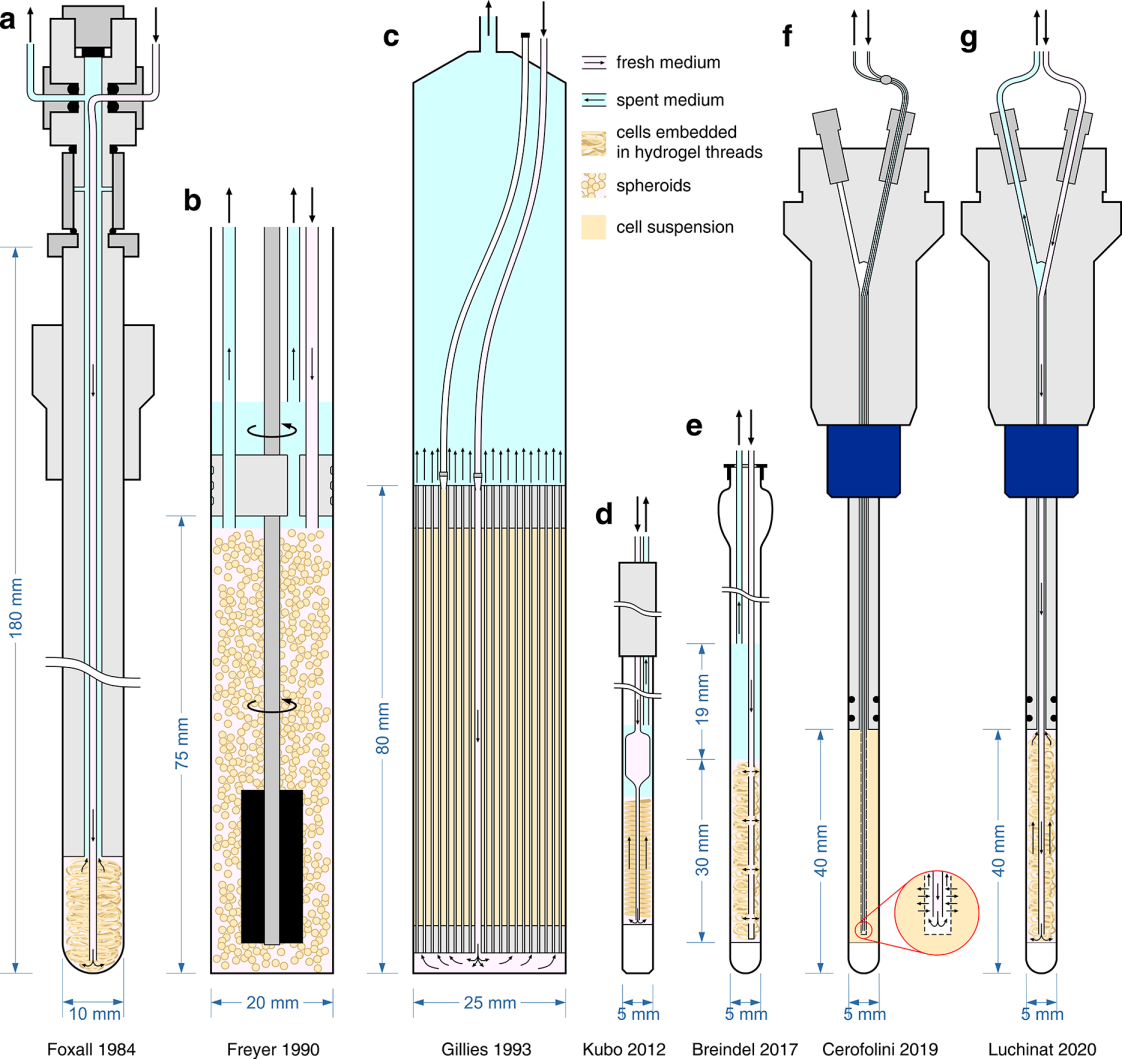
Schematics of wide-bore (a–c) and modern narrow-bore (d–g) NMR bioreactors for mammalian cells. (a) Perfusion bioreactor by Foxall et al.;^287^ cells are embedded in low-gelling agarose. (b) Stirred bioreactor by Freyer et al.;^290^ spheroids are kept in suspension by mechanical stirring. (c) Hollow-fiber reactor by Gillies et al.;^292^ cells growing in suspension are directly inoculated, whereas cells growing in adhesion are embedded in collagen beads prior to inoculation. (d) Perfusion bioreactor by Kubo et al.;^35^ cells are embedded in Mebiol gel. (e) bioreactor by Breindel et al.;^164^ cells embedded in low-gelling agarose are perfused with a horizontal drip irrigation system. (f, g) Two alternative bioreactor setups based on a sealed flow unit; (f) cells are kept as a suspension and the nutrients diffuse through a coaxial microdialysis membrane (Cerofolini et al.^294^); (g) cells are embedded in low-gelling agarose and perfused as in (a, d) (Luchinat et al.^171^). Schematics were redrawn to scale based on the technical details and illustrations reported in the works cited above.

For mammalian cells, which typically grow in adhesion as a monolayer or in multicellular 3D structures such as spheroids, different approaches were necessary. The Edelman group employed a perfusion system to analyze mammalian cells grown as monolayers.^286^ Mouse embryo fibroblasts were grown on the surface of microcarrier beads, which were placed in a 15 mm NMR tube and perfused with a fresh medium to maintain steady-state conditions during the analysis. This study demonstrated that NMR bioreactors could also be applied to anchorage-dependent cells. A different approach consisted of encapsulating the cells in threads or beads by using gel matrices.^287,288^ Foxall et al. applied this strategy to encapsulate yeast cells and Chinese hamster lung fibroblasts (CHLF).^287,289^ In the latter work, cells were encapsulated by forcing a mixture of low-gelling agarose matrix and CHFL cells through a cooled Teflon tube with an air pressure jet. The formed threads were collected in a 10 mm NMR tube, which was perfused with a fresh medium using a peristaltic pump connected to a warmed medium reservoir (Figure 7a). Narayan et al. followed a similar approach, by embedding PC-3 human carcinoma-derived cells within calcium alginate beads. The NMR analysis was performed in a 20 mm NMR tube perfused with a fresh medium.^288^ Freyer et al. developed a system for keeping viable EMT6/Ro mouse mammary carcinoma-derived spheroids for long-time NMR experiments.^290^ A pump system was employed for perfusing a suspension of stirred spheroids with a complete prewarmed and oxygenated medium. The perfusion chamber was built around a flat-bottomed 20 mm NMR tube and was mechanically stirred and provided with outlets to allow the circulation of the medium (Figure 7b). A markedly different type of NMR bioreactor for growing adherent cells in the NMR spectrometer made use of hollow-fiber reactors.^291,292^ In these devices, cells are inoculated in a 25 mm wide cylindrical chamber that contains cellulose acetate/cellulose nitrate hollow fibers with a high porosity (Figure 7c). A growth medium is flowed continuously through the fibers, where it diffuses through and reaches the cell chamber where the cells are confined, supporting cell growth at high densities. Gillies et al. took additional efforts to develop an advanced supporting circuit outside of the magnet to maintain the proper medium composition in terms of pH, chemical composition, and dissolved gases.^292^

As mentioned earlier, despite the advancements in NMR bioreactor design, a new generation of NMR bioreactors had to be (re)invented to fit modern narrow-bore magnets for in-cell NMR applications. Three main concepts from the previous designs have currently been explored in the development of modern NMR bioreactors: a circulating encapsulated cells (CEC) bioreactor,^293^ a continuous-flow system that employs immobilized cells,^35,50,164,171^ and a membrane perfusion system to provide a fresh medium to the cells suspended in the NMR tube.^294^

The CEC bioreactor concept was developed by the Pielak group and is one of a kind, although it borrows concepts from the stirred cellular suspension used by Freyer et al.^290^ The CEC system employs a perfusion pump to both stir and supply a fresh medium to cells electronically encapsulated into 1 mm diameter Ca^2+^ alginate spheres.^293^ Unlike the previous systems, however, the CEC bioreactor does not operate in a continuous mode: when the pump is active the alginate embedded cells are pushed in the circulation chamber where the exchange of oxygen and nutrients occurs; when the pump is switched off, the cells fall back into the detection region allowing one to acquire NMR spectra. After 18 h of experiments, in which Sharaf et al. monitored the α-synuclein overexpression in *E. coli*, the cell viability was estimated to be 95%, and the pH of the medium remained at 7.0 for the entire duration of the measurements.

Bioreactors developed later for in-cell NMR were designed for continuous operation. The most commonly implemented design is based on the continuous perfusion of gel-encapsulated cells, similar to the previously mentioned approaches.^287,288^ In these bioreactors, cells are encapsulated in various gel matrices and confined in the NMR tube, where a constant flow of fresh medium is applied to allow the exchange of nutrients, metabolites, and gases. Different gel matrices have been employed for this purpose. Kubo et al. first implemented such a design making use of Mebiol gel, a copolymer of poly(*N*-isopropylacrylamide) and poly(ethylene glycol) that becomes a gel when heated.^35^ HeLa cells mixed with a Mebiol solution are transferred to a 5 mm Shigemi NMR tube, which is quickly warmed to 37 °C to allow a sol–gel transition, encapsulating the cells in a coil-shaped thread (Figure 7d). A series of ^31^P NMR spectra showed that, after 5 h of measurement, the setup allowed the intracellular ATP concentration to remain stable for up to 5 h, while after 15 h the cell viability decreased below 80%. Conversely, in the absence of flow, a complete depletion of ATP occurred after 30 min, and less than 20% of cells remained viable after 15 h. Similar NMR bioreactors were later implemented following the same approach but making use of low-gelling agarose to encapsulate cells in a gel thread. Breindel et al. proposed a pumpless setup where a constant flow of medium is allowed by a gravity siphon. Unlike other setups, the inlet is sealed at the end and pierced with 50 μm wide holes to create a horizontal “irrigation” system (Figure 7e). The device was applied to bacteria and HeLa cells, both encapsulated in agarose threads, ensuring a stable metabolic activity over 24 h.^164^ Carvalho et al. implemented a similar design by using peristaltic pumps for both the inlet and the outlet flux, where HeLa cells embedded in agarose threads were confined in the tube using a Teflon plug. Cell viability was shown to be preserved for up to 16 h.^295^ Luchinat et al. employed a commercially available sealed flow unit to implement an analogous bioreactor setup. The sealing of the 5 mm flow tube through a series of o-rings allowed one to confine HEK293T cells embedded in agarose threads in the bioreactor, while a nutrient flow was ensured by an FPLC pump (Figure 7g). The device preserved greater than 90% cell viability and sustained metabolic activity for up to 72 h.^171^ In most of the bioreactor setups described above, the correct positioning of the agarose thread in the detection zone of the NMR tube is ensured by filling the bottom of the tube with agarose gel, creating a Shigemi-like shape.

Lastly, a third type of bioreactor suitable for suspended cells was developed by Cerofolini et al.^294^ This setup used the sealed flow unit described above, in which mammalian cells were kept confined as a suspension in a growth medium containing 30% Percoll. The inlet of the flow unit was replaced with a microdialysis membrane with a cutoff of 1 MDa. Within the membrane, the nutrients flowed at the bottom through a coaxial inlet and reached the cell suspension by diffusing across the membrane, while bioproducts diffused in the opposite direction into the membrane and were removed through a coaxial outlet (Figure 7f). The metabolic activity was measured by ^1^H and ^31^P spectra and was maintained constant for up to 13 h. This design exploits a concept similar to that of the hollow fiber bioreactor of Gonzales-Mendez et al. and Gillies et al.,^291,292^ although the use of a single microdialysis membrane (instead of many hollow fibers) decreases the surface-to-volume ratio and likely reduces the overall efficiency of the nutrient/byproduct exchange.

The 5 mm wide bioreactors described above allowed one to keep a high number of cells, both bacterial and human, alive and metabolically active for several hours/days, enabling the acquisition of more complex and informative in-cell NMR spectra and making it possible to monitor time-dependent phenomena at the structural level. However, it is striking to note how advanced the external support systems of the older devices were, compared with the modern bioreactors, in terms of control of pH, CO_2_, and chemical composition. Thus, the next generation of NMR bioreactors should focus more on the optimization of cell-growth conditions, by implementing external systems for a growth medium control and by using appropriate scaffolds for culturing cells in-magnet.

### 4.2 Bioreactor Applications

One straightforward application of bioreactors is the acquisition of longer in-cell NMR experiments. This improves the sensitivity of the methodology^35^ and is crucial for an NOE-based protein structure determination in eukaryotic cells, where recording long 3D experiments would be impractical due to the short sample lifetime (see section 3.3).^73^ In addition, bioreactors offer the unique possibility of observing cellular events in real time by NMR. Indeed, not only can they maintain cells in a stable metabolic state but also allow one to change such a state in a controlled manner, for example, by adding drugs or metal ions. Therefore, bioreactors are ideally applied to monitor in real time the concentration of cellular metabolites and their change from normal to stress conditions and to observe how a protein conformation changes upon interaction with cofactors, drugs, and other proteins.

#### 4.2.1 Cellular Metabolism

Since the development of the first NMR bioreactors, the main application was the study of metabolic activity in living cells. The use of ^31^P NMR enables the detection and quantification of various metabolites such as ATP, ADP, NAD(P)H, and inorganic phosphate, in addition to other polyphosphates, phosphomonoesters, and sugar phosphates.^282^^13^C detection NMR was used to monitor different metabolic pathways, by analyzing the different build-up and degradation rates of ^13^C-labeled substrates in real time.^284^ Hartbrich et al. monitored the glucose consumption of *Zymomonas mobiliz* through ^31^P NMR experiments, from which they identified a cyclic pyrophosphate metabolite that tended to decompose in classical in vitro experiments and measured the conversion of ^13^C glucose by *Corynebacterium glutamicum*, where they determined the flux distributions over the two metabolic pathways of lysine biosynthesis by analyzing the different build-up and degradation rates of ^13^C-labeled l-lactate, l-glutamate, l-lysine, and succinate.^284^ Similar experiments were performed by Majors et al. on *Eubacterium aggregans.* This microorganism was maintained in a controlled grow condition, and through 1D ^1^H spectra, it was possible to monitor the utilization of glucose and fructose and consequently different byproduct excretions like formate, pyruvate, acetate, lactate, ethanol, and *n*-butyrate.^286^ Real-time ^31^P NMR has been extensively used to monitor changes in the cellular energetic levels upon normal or stressed conditions, to assess the ensuing metabolic changes and the overall cell viability in the bioreactor systems.^282,286,290,292^ A series of works focused on the unicellular seaweed *Dunaliella salina* evaluated how osmotic shocks could influence the energetic metabolism.^296,297^^31^P NMR was also employed to evaluate the energetic behavior of *E. coli* cells in both aerobic and anaerobic growth conditions.^283^ The analysis showed that the nucleoside triphosphates and inorganic phosphate levels decreased in anaerobic conditions and returned to standard values after the aerobic condition was restored.

**Figure 8:**
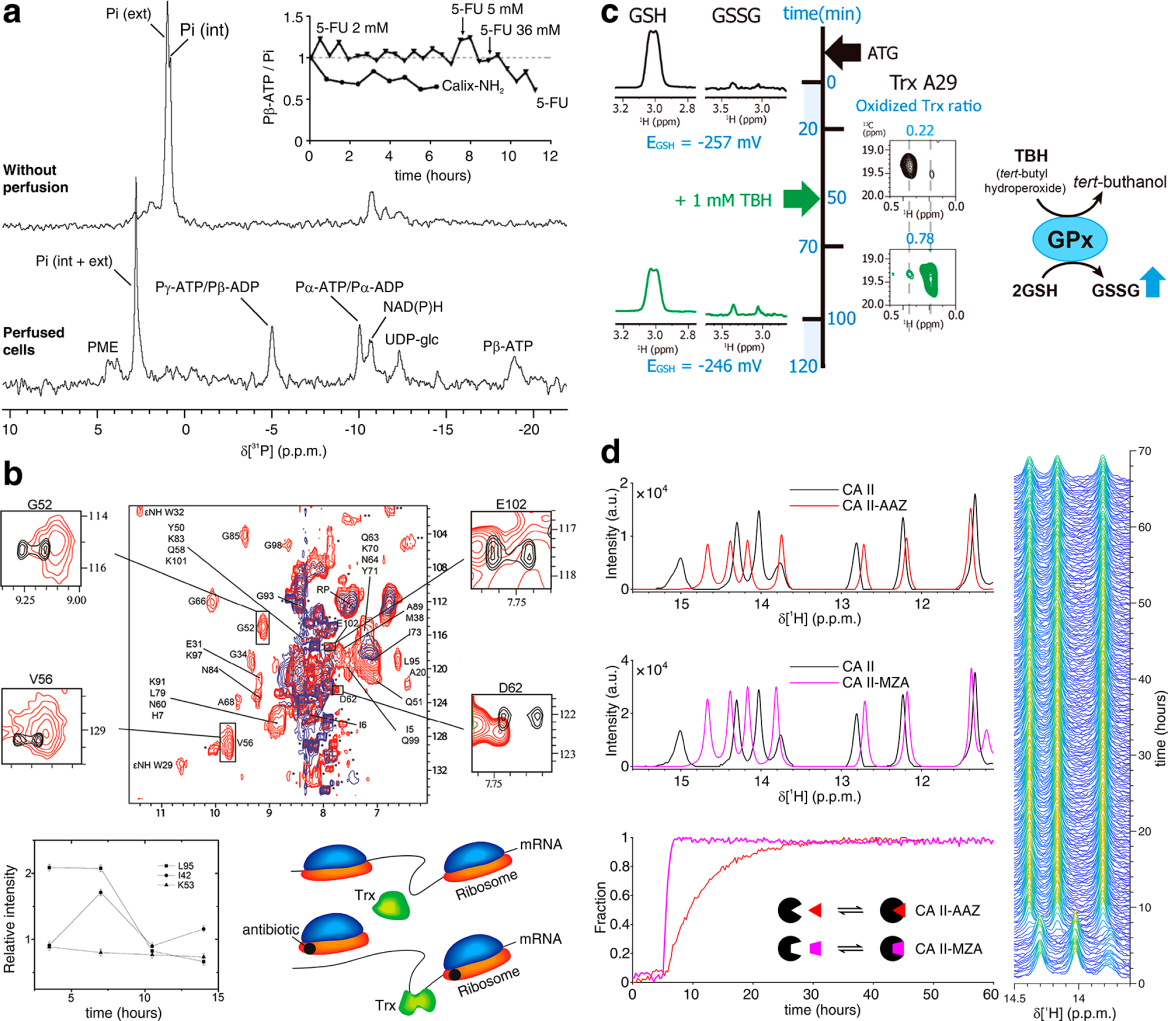
Application of bioreactors to real-time in-cell NMR. (a) Analysis of the cellular metabolic state: ^31^P NMR spectra of HeLa cells in the absence (top) and in the presence (bottom) of a flow of nutrients. (inset) Monitoring the ratio between P_β_-ATP and inorganic phosphate (P_i_) as a function of time reveals a decrease of cellular metabolic activity upon treatment with cytotoxic compounds. Reprinted from Carvalho et al.,^295^ with permission from Elsevier. (b) Changes of protein quinary structure upon antibiotic binding to the ribosome: (top) overlay of the in-cell ^1^H–^15^N NMR spectra of thioredoxin (Trx) in *E. coli* cells before (red) and after (blue) treatment with tetracycline; changes in Trx peak intensities as a function of time (bottom left); possible mechanism of how an antibiotic binding to the ribosome induces Trx-mRNA interactions (bottom right). Reprinted with permission from Breindel et al.^164^ Copyright 2017 American Chemical Society. (c) Changes in protein and glutathione redox state: real-time in-cell NMR spectra of Trx and glutathione in HeLa cells after treatment with ATG, a thioredoxin reductase inhibitor (black), and the oxidant *tert*-butyl hydroperoxide (TBH, green). Reprinted with permission from Mochizuki et al.^36^ Copyright 2018 American Chemical Society. (d) Monitoring protein–ligand interactions in real time: reconstructed ^1^H NMR spectra of carbonic anhydrase II (CA II) in HEK293T cells before (black) and after treatment with acetazolamide (AAZ, red) or methazolamide (MZA, magenta); concentration profiles of ligand-bound CA II as a function of time (bottom); example of a time series of raw in-cell ^1^H NMR spectra (right). Reprinted with permission from Luchinat et al.^171^ Copyright 2020 American Chemical Society.

Drugs are known to influence metabolic pathways by acting on different targets in human cells. Carvalho et al. employed the bioreactor to evaluate the cytotoxic effects of pharmacological compounds on the viability of HeLa cells.^296^ The cytotoxic effect of two selected drugs, Calix-NH_2_ and 5-FU, was assessed by ^31^P NMR. The drugs were separately added in the perfusion medium at different concentrations, and the energy storage level of the cells was monitored (Figure 8a). The results showed that both drugs were able to reduce the ATP/P_i_ ratio, with negligible effects on the intracellular pH. The above work showed how bioreactors allow evaluating real-time changes in the metabolome of human living cells. Real-time recording of 2D, or 1D isotope-filtered, heteronuclear NMR spectra can provide information about the metabolic state of cells following a pharmaceutical treatment, using ^13^C_6_-labeled glucose or other metabolites as a tracer, as shown by Wen et al. and Alshamleh et al.^298,299^

Perfusion bioreactors have also been combined with tracer-based ^13^C NMR to monitor the kinetics of cellular metabolism in a real-time fashion. Typically, however, the sensitivity limit of ^13^C NMR spectroscopy does not allow a temporal resolution in the order of seconds, which is required for enzyme kinetics studies in cells. Dissolution DNP (dDNP), which is traditionally applied to metabolic imaging in vivo,^300^ has been shown to greatly increase the sensitivity of such approaches also for studies on cell cultures. By modifying a perfusion bioreactor design previously developed by Degani et al.,^301^ Frydman and Degani developed an injection/perfusion system for investigating the metabolism of hyperpolarized ^13^C pyruvate in human breast cancer cells by dDNP-enhanced NMR.^302^ The kinetics of pyruvate-to-lactate conversion were characterized, by reliably determining the Michaelis–Menten characteristics of the reaction under various cell stress conditions. The same approach was later employed to characterize the metabolism of hyperpolarized ^13^C,^2^H-glucose in breast cancer cells, where the dDNP enhancement allowed the detection of several glycolysis intermediates.^303^ The Macdonald group developed a similar device for dDNP-enhanced NMR of ^13^C pyruvate, consisting of a fluidized-bed bioreactor in which rat hepatoma encapsulated in alginate beads was perfused at high flow rates to prevent a packing of the beads.^304,305^ Furthermore, the high sensitivity of dDNP allows for a fast data acquisition, thus making it possible to study metabolic fluxes in cells kept in suspension over the course of a few minutes, without the need for NMR bioreactors to ensure cell viability.^306−311^

#### 4.2.2 Real-Time In-Cell NMR

One of the most advanced applications of “modern” NMR bioreactors focuses on the real-time monitoring of proteins involved in intracellular processes. The Pielak group applied the CEC bioreactor to monitor over time the expression of α-synuclein inside alginate-encapsulated *E. coli* cells. A series of 2D ^1^H–^15^N spectra was acquired before and after the induction, alternated with pause times during which the pump was switched on to replenish fresh nutrients. The signals of α-synuclein increased over time until they reached an ∼0.8 mM intracellular concentration after 18 h.^293^ The Shekhtman group applied the continuous-flow bioreactor to show that antibiotics inhibiting the bacterial ribosome can alter the quinary interactions of the bacterial thioredoxin (Trx), which was previously shown to change its quinary structure upon interaction with mRNA (see section 3.1).^47^ A series of ^1^H–^15^N spectra was recorded in the absence of antibiotics and following a treatment with tetracycline or streptomycin. An SVD analysis (see section 2.3) highlighted changes in the Trx quinary interactions induced by the antibiotics, suggesting that the latter compete directly with Trx for binding to RNA (Figure 8b).^164^

In a study of human cells, Shimada and Nishida applied bioreactor-assisted real-time in-cell NMR to monitor the intracellular redox state of human Trx and glutathione in response to oxidative stress in a time-resolved fashion (see section 3.4.2).^36^ The use of a bioreactor allowed them to assess the redox state of Trx and glutathione in real time, in the presence of oxidative stress-inducing agents, by recording a series of ^1^H–^13^C spectra to detect signals from both [^13^CH_3_]Ala-labeled Trx and [^13^C]Cys-labeled glutathione (Figure 8c). More recently, the same group investigated, by real-time in-cell NMR, the GTPase cycle of wild-type and oncogenic mutants of HRAS in human cells.^37^ HRAS is part of the small guanosine phosphatase proteins that, after the stimulation of the tyrosine kinase receptors, induces cell proliferation, motility, and survival pathways. RAS proteins exist in two main states, the inactive GDP-bound state and the active GTP-bound state. The transition between the two states causes structural conformational changes that allow the protein to interact with the downstream effectors.^312^ HRAS mutations that lead to the aberrant activation of the protein are frequently found in different types of cancer,^313^ thus making HRAS a relevant drug target. The levels of GDP- and GTP-bound HRAS were measured in real time from a series of ^1^H–^13^C spectra on [^13^CH_3_]isoleucine-labeled HRAS delivered to HeLa cells. It was found that GTP-bound RAS, both wild-type and the oncogenic mutants, is lower than in vitro due to an increase of the GTP hydrolysis rate and a decrease of the GDP-GTP exchange rate. Furthermore, experiments in-lysate and in vitro in crowded, viscous solutions revealed that, while the increased hydrolysis rate is caused by specific cytoplasmic macromolecules, the decreased exchange rate is caused by the higher viscosity—rather than the crowding—of the cellular environment.^37^ Our group applied the NMR bioreactor to monitor in real time the drug binding to carbonic anhydrase II (CA II) and the ebselen-mediated cysteine oxidation of superoxide dismutase 1 (SOD1).^171^ The binding of the approved drugs acetazolamide (AAZ) and methazolamide (MZA) to CA II, which was previously observed by “static” in-cell NMR,^92^ was monitored as a function of time by measuring the fractions of free and bound protein in a series of 1D H^1^ NMR spectra (Figure 8d). Time-dependent binding curves of both AAZ and MZA were obtained by a quantitative analysis of the NMR data by MCR-ALS (see section 2.3). The results were consistent with the different cell membrane permeability of the two drugs observed previously. The same approach was applied to monitor the formation of SOD1 intramolecular disulfide bond catalyzed by ebselen, which was previously shown to stabilize fALS-linked mutants of the protein in human cells,^224^ from a series of 2D ^1^H–^15^N spectra.

The above works show that NMR bioreactors applied to in-cell NMR open up new and previously unthinkable possibilities to study in real time chemical and structural changes involving macromolecules in the cellular context, with important applications to cellular/structural biology and drug development.

## 5 Future Perspectives

The works outlined in the previous sections provide an overview of the methodological advancements and applications of in-cell solution NMR in the last ∼20 years. We first reviewed the state of the art of the methodology, focusing especially on the approaches for NMR analysis in mammalian cells developed in past decade. We then overviewed the main fields of application of in-cell NMR, highlighting some of the most important findings made possible by this approach. Lastly, we focused on the parallel development of NMR bioreactors, needed to increase the lifetime of the cells, and on their application to the real-time NMR analysis of living cells.

Overall, the works reviewed above highlight the great potential of in-cell NMR to investigate structural and functional aspects of macromolecules in living cells, providing unique insights on their complex interplay with the other components of cellular milieu. Despite the increased efforts required for characterizing molecules in cells compared to an in vitro analysis, which, it is worth noting, still constitutes a fundamental part of the research, we believe that, in the long term, in-cell NMR as well as other cellular structural approaches will be the key to answer fundamental biological questions. A few examples of such potential include the finding that single residues on the surface of folded proteins can greatly affect the intracellular folding stability and the rotational diffusion, due to the strong electrostatic interactions with other cellular components that ultimately underlie the quinary structure of the protein;^46,90,143,144,178,179,183−185^ the ability to determine which metalation and cysteine redox states of a protein—equally possible in vitro—are compatible with the cellular metal and redox homeostasis and how they are influenced by the presence of specific partners;^36,57,84,87−89,228^ the settling of a debate on the ensemble of conformational states adopted by α-syn in the cytoplasm thanks to the detailed characterization provided by NMR relaxation, solvent exchange, and chemical shift analysis in bacteria and human cells;^39,58,146,165^ the demonstration that certain short DNA sequences, i-motifs, can form stable structures in the nuclear environment of human cells.^105^

Undoubtedly, these conclusions would have been (almost) impossible to draw without resorting to in-cell NMR. However, as it often happens with novel methodologies, after the high expectations raised by the first landmark developments were not fully met in the following years, the scientific community—especially the NMR community—approached the methodology with increased skepticism. This is partly due to some intrinsic limits of the methodology, which perhaps have not been stated clearly enough in the beginning by those, including us, trying to highlight the strengths of the in-cell approach.

The most critical limitation, which no NMR hardware development can overcome, is the line broadening beyond detection suffered by some (many?) proteins. It is now clearly demonstrated that such a broadening is the extreme consequence of the slow tumbling caused by attractive interactions with high-molecular-weight cellular components. Paradoxically, the quinary structure of proteins, which was finally decoded thanks to in-cell NMR, makes in-cell NMR impossible! The solution to this paradox is to forego the notion that soluble proteins that interact too strongly with large cellular structures are to be treated as solutes. Instead, they should be thought of as “solid-like” entities and, therefore, are more properly characterized by cellular solid-state NMR. While outside the scope of this Review, cellular solid-state NMR approaches have been developed in the past decade in parallel to in-cell solution NMR, and they have been shown to provide atomic-level structural and functional insights on macromolecules in intact cellular settings under freezing or cryogenic conditions.^48,314−316^

Another limitation of in-cell NMR is linked to the intrinsically low sensitivity of NMR. This does not strictly prevent NMR detection but, in practice, imposes higher thresholds of macromolecule concentration required to perform NMR experiments in a time frame compatible with the lifetime of the cell sample. Hence, it is required to artificially increase the intracellular levels of a macromolecule, compared to the endogenous ones, at the risk of introducing artifacts due to the nonphysiological intracellular concentration of molecule. Notably, the detection limit is highly dependent on the properties of the molecule under study, as its average tumbling rate hugely affects line broadening and therefore sensitivity. Without resorting to bioreactors to increase the sample lifetime, it has been shown that IDPs can be observed in viable human cells down to an effective concentration of ∼15 μM,^39^ whereas folded proteins between 10 and 40 kDa require higher concentrations, typically in the 50–200 μM range, depending on the extent of their interactions with the cellular milieu. NMR hardware improvements, such as higher magnetic fields and last-generation electronics, have further contributed to lower the detection limit of in-cell NMR over the years (ref (1).2 GHz). Although certainly beneficial, the incremental progress of NMR hardware will not enable NMR detection at orders-of-magnitude lower concentrations. While the sensitivity of solid-state NMR can be boosted by resorting to DNP, dDNP polarization enhancement strategies for solution NMR are limited to small molecules and, as such, they have found widespread application in the analysis of intracellular metabolic fluxes,^302,303^ but to the best of our knowledge they have not been applied to study macromolecules in intact cells.

NMR bioreactors can partly overcome the sensitivity issue, because they are able to increase the useful time frame for acquisition of NMR spectra without sacrificing cell viability. Indeed, the latest iteration of bioreactor setups allows one to keep a high number of cells viable for up to 72 h, an ∼36-fold increase in acquisition time compared to the same cells under “static” conditions, which, when translated to a signal-to-noise ratio, amounts to an approximately sixfold increase, much higher than the gain from NMR hardware that can be reasonably foreseen in the near future. Furthermore, longer acquisition times in the bioreactor enable one to monitor intracellular processes as they occur in real time. In light of the great potential of bioreactors to mitigate the sensitivity limitation and to monitor time-dependent processes, we expect that more advanced bioreactors will be developed that will make use of improved materials for supporting cell viability and will provide a finer control of the cell culture conditions.

Concerning the future applications of in-cell NMR, many research areas will gain precious insight from NMR studies of macromolecules performed on living cells, thanks to the unique kind of information provided. In our view, future applications should include (but not be limited to): a deeper study of the biological role of quinary interactions, to understand whether they are truly nonspecific or if instead there are some key effectors (such as ribosomes, or mRNAs) responsible for the most part of these interactions; a more systematic study of the conformational dynamics of intrinsically disordered regions of proteins, which make up a huge, underrepresented part of the known proteome (almost one-half of the proteins encoded by the human genome contains a disordered segment),^317^ with a specific focus on those known to be involved in diseases; the investigation by real-time NMR of the initial steps of the misfolding of proteins involved in degenerative diseases, to understand how the cellular environment affects such a process and which are the structural and functional properties of the misfolded species.

Future research in the above areas will certainly benefit from the combination of in-cell NMR with other methodologies capable of providing complementary information in the same cellular settings. Cryo-electron tomography^318^ and optical fluorescence microscopy (including super-resolution techniques^319^ and FRET- or lifetime-based imaging/spectroscopy^320^) can provide insights into the structure and dynamics of intracellular macromolecules and complexes with extremely high spatial resolution, down to the sub-nanometer range, which is not accessible to an ensemble-based methodology like NMR spectroscopy. Other complementary cell-based imaging approaches include mass spectrometry imaging^321^ and X-ray fluorescence microscopy,^322^ which provide the spatial distribution of cellular metabolites and metal ions, respectively (integration of in-cell NMR with the latter was shown by Luchinat and Banci^323^).

Finally, we envision a more widespread application of in-cell NMR to the field of drug development, where it can bridge the gap between the in vitro characterization/optimization of lead compounds and the cellular assays that often precede the use of preclinical models. While cell-based assays for some types of drugs are well-established (e.g., proliferation assays for anticancer drugs), with such assays it is often nontrivial, or impossible, to demonstrate an actual target engagement for the screened compounds, resulting in promising candidate drugs that may not actually bind to the intended target, increasing the risk of failure in the preclinical or clinical phases due to toxicity or lack of efficacy. To this aim, while protein-based real-time NMR screening has been recently successfully applied, new approaches will have to be developed to monitor by real-time NMR the fate of a (nonmodified) drug as it penetrates the cells, diffuses into different compartments, and finally either binds to its intended target or, most importantly, interacts with other molecules.

Over the last two decades, in-cell NMR has affirmed itself as an exciting new branch of biomolecular NMR. New approaches have been steadily developed, which have extended the applicability of the methodology to increasingly complex types of cells. After much initial interest and some skepticism, perhaps due to exceedingly high expectations, in-cell NMR has shown its capabilities to answer challenging biological questions that arise from the complexity of the cellular environment, and we firmly believe that it will continue to do so and will be developed further and be applied to many research areas of Life Sciences.
